# SmSP2: A serine protease secreted by the blood fluke pathogen *Schistosoma mansoni* with anti-hemostatic properties

**DOI:** 10.1371/journal.pntd.0006446

**Published:** 2018-04-20

**Authors:** Adrian Leontovyč, Lenka Ulrychová, Anthony J. O’Donoghue, Jiří Vondrášek, Lucie Marešová, Martin Hubálek, Pavla Fajtová, Marta Chanová, Zhenze Jiang, Charles S. Craik, Conor R. Caffrey, Michael Mareš, Jan Dvořák, Martin Horn

**Affiliations:** 1 Institute of Organic Chemistry and Biochemistry, The Czech Academy of Sciences, Prague, Czech Republic; 2 First Faculty of Medicine, Charles University, Prague, Czech Republic; 3 Department of Parasitology, Faculty of Science, Charles University, Prague, Czech Republic; 4 Center for Discovery and Innovation in Parasitic Diseases, Skaggs School of Pharmacy and Pharmaceutical Sciences, University of California San Diego, La Jolla, CA, United States of America; 5 Institute of Immunology and Microbiology, First Faculty of Medicine, Charles University and General University Hospital in Prague, Prague, Czech Republic; 6 Department of Pharmaceutical Chemistry, University of California San Francisco, San Francisco, CA, United States of America; 7 Department of Zoology and Fisheries, Faculty of Agrobiology, Food and Natural Resources, Czech University of Life Sciences Prague, Prague, Czech Republic; University of Pennsylvania, UNITED STATES

## Abstract

**Background:**

Serine proteases are important virulence factors for many pathogens. Recently, we discovered a group of trypsin-like serine proteases with domain organization unique to flatworm parasites and containing a thrombospondin type 1 repeat (TSR-1). These proteases are recognized as antigens during host infection and may prove useful as anthelminthic vaccines, however their molecular characteristics are under-studied. Here, we characterize the structural and proteolytic attributes of serine protease 2 (SmSP2) from *Schistosoma mansoni*, one of the major species responsible for the tropical infectious disease, schistosomiasis.

**Methodology/Principal findings:**

SmSP2 comprises three domains: a histidine stretch, TSR-1 and a serine protease domain. The cleavage specificity of recombinant SmSP2 was determined using positional scanning and multiplex combinatorial libraries and the determinants of specificity were identified with 3D homology models, demonstrating a trypsin-like endopeptidase mode of action. SmSP2 displayed restricted proteolysis on protein substrates. It activated tissue plasminogen activator and plasminogen as key components of the fibrinolytic system, and released the vasoregulatory peptide, kinin, from kininogen. SmSP2 was detected in the surface tegument, esophageal glands and reproductive organs of the adult parasite by immunofluorescence microscopy, and in the excretory/secretory products by immunoblotting.

**Conclusions/Significance:**

The data suggest that SmSP2 is secreted, functions at the host-parasite interface and contributes to the survival of the parasite by manipulating host vasodilatation and fibrinolysis. SmSP2 may be, therefore, a potential target for anti-schistosomal therapy.

## Author summary

Schistosomiasis (bilharziasis) is a global parasitic infection with more than 240 million people infected. It is caused by *Schistosoma* flatworms that live in the bloodstream. Current treatment relies on one drug, and no effective vaccine has yet been developed. Proteolytic enzymes (proteases) help the parasite survive in the mammalian host, allowing the schistosome to invade, feed, grow, reproduce and, manipulate the immune system. Thus, proteases are considered potential drug and vaccine targets. We previously described, and herein, investigate at the protein level, SmSP2, the major serine protease produced by the blood-dwelling stages of *S*. *mansoni*. We show that SmSP2 is secreted by the parasite and effectively processes host blood bioactive peptides and proteins that are involved in fibrinolysis and regulation of vascular tone. We propose, therefore, that SmSP2 modulates host hemostasis to promote parasite infection and survival. Thus, SmSP2 and similar proteins in other parasitic flatworms represent potential targets for novel drug or vaccine interventions.

## Introduction

Schistosomiasis (bilharziasis) is a chronic infectious disease caused by a trematode blood flukes of the genus *Schistosoma*. A public health problem in 74 developing countries, the parasite infects over 240 million people with up to 700 million people at risk [1, 2]. Adult schistosomes can reside for decades in the host vascular system as male-female pairs producing hundreds of eggs per day [3]. Morbidity arises from immuno-pathological reactions to and entrapment of schistosome eggs in various tissues [4]. In the absence of a vaccine, treatment and control of schistosomiasis relies on a single drug, praziquantel (PZQ) [5, 6]. During its complex life cycle, the parasite survives in various environments by presenting or releasing bioactive molecules that aid survival and modulate host physiology [7–10]. Disruption of these potential mechanisms by specific drugs or vaccines may provide therapeutic benefits.

Proteolytic enzymes (proteases) of schistosomes [11] are attractive drug targets as they frequently operate at the host–parasite interface, and facilitating parasite invasion, migration, nutrition and immune evasion [12–14]. Most studies on schistosome proteases, to date, have focused on cysteine and aspartic proteases that contribute to the digestion of the blood meal [15, 16]. Among them, the cathepsin B1 of *S*. *mansoni* (SmCB1) has been validated in a murine model of schistosomiasis as a chemotherapeutic target [17], and small molecule inhibitors of SmCB1 are under investigation as potential drug leads [18–20].

Schistosome serine proteases (SPs) are less studied with the exception of cercarial elastase, which facilitates penetration of the human host by infective larvae [21, 22]. Recently, we uncovered a repertoire of *S*. *mansoni* trypsin- and chymotrypsin-type S1 family serine proteases (SmSPs) by employing a series of genomic, transcriptomic, proteolytic and phylogenetic investigational strategies [23]. Among these, SmSP2 is the most abundantly expressed in blood-dwelling stages [23]. Interestingly, SmSP2 ortholog (mastin) was identified as potential vaccine targets in *Schistosoma haematobium* based on IgG1 immune response of individuals with drug-induced resistance [24].

In this study, we report the first detailed biochemical and enzymatic characterization of SmSP2. This enzyme processes several proteins and peptides that are involved in host proteolytic cascades, *i*.*e*., blood coagulation, fibrinolysis and blood pressure regulation, and, thus, may interfere with critical vascular hemostatic processes. Accordingly, SmSP2 may be a target for novel anti-schistosomal therapeutics.

## Materials and methods

### Ethics statement

Research with experimental animals was performed in accordance with the animal welfare laws of the Czech Republic and in accordance with European regulations for transport, housing and care of laboratory animals (Directive 2010/63/EU on the protection of animals used for scientific purposes). The project of experiments including the use of experimental animals for present study was approved by Ministry of education, youth and sports of the Czech Republic (approval number MSMT—7063/2017-2). All the animals used in the study were maintained by certified person (certificate number CZ 02627) in specifically accredited laboratories of Institute of Immunology and Microbiology of the First Faculty of Medicine, Charles University and the General University Hospital in Prague (accreditation number 70030/2013-MZE-17214), both issued by the Ministry of Agriculture of the Czech Republic.

### Schistosome material

*Schistosoma mansoni* (a Puerto Rican isolate) was routinely maintained in the laboratory by cycling between the intermediate snail host, *Biomphalaria glabrata*, and outbred ICR mice as definitive hosts. Infective larvae (cercariae) were released after light stimulation from infected snails placed in fresh water. Adult mice were infected by immersing the feet and tails for 45 min in 50 ml of water containing approximately 500 cercariae. Six weeks post infection, mice were euthanized by an intra-peritoneal injection of ketamine (Narkamon 5% - 1.2 mL/kg bw) and xylazine (Rometar 2% - 0.6 mL/kg bw) and the worms recovered from the portal vein by transcardial perfusion with RPMI 1640 medium as described previously [25–27]. Newly transformed schistosomula (NTS) were prepared by mechanically transforming cercariae and cultivated in a Basch Medium 169 [28] containing 5% fetal calf serum, 100 units/mL penicillin and 100 μg/mL streptomycin for 5 days at 37°C under a 5% CO2 atmosphere.

### Preparation of schistosome extract, excretory/secretory products collection and purification of native SmSP2

Soluble protein extracts (1–3 mg protein/mL) from *S*. *mansoni* adults were prepared by homogenization in 50 mM Tris-HCl, pH 8.0, containing 1% CHAPS, 1 mM EDTA, 1 μM pepstatin and 1 μM E-64 on an ice bath. The extract was cleared by centrifugation (16,000 *g* at 4°C for 10 min), ultra-filtered using a 0.22 μm Ultrafree-MC device (Millipore) and stored at -80°C. Excretory/secretory products (ESP) of adult worms were collected as described previously [29]. Specifically, fifty pairs of adult worms were washed five times in Basch medium 169 containing 5% fetal bovine serum, 100 U/mL penicillin, 100 μg/mL streptomycin and 1% Fungizone (Gibco), incubated for 1h at 37°C under a 5% CO_2_ atmosphere, washed five times and then incubated overnight at 37°C in 5% CO_2_ in the above medium supplemented with 5% fetal calf serum but in the absence of Fungizone. Parasites were washed three times in the above medium and then washed 10 times in M-199 medium containing 100 U/ml penicillin and 100 μg/ml streptomycin, but without serum. Adults were evenly distributed in 5 ml of the same medium in a 6-well cultivation dish and incubated for 16 h at 37°C in 5% CO_2_. Medium containing ESP was removed, filtered over an Ultrafree-MC 0.22 μm filter (Millipore), buffer exchanged into ice-cold PBS, concentrated to 2 mL using Amicon Ultracel-10K filters (Millipore) and aliquots stored at -80°C.

Native SmSP2 was purified from the adult schistosome extract using Ni^2+^ chelating chromatography (Hi-Trap IMAC FF column, GE Healthcare Life Sciences) under native conditions. The bound material was eluted using a 0.5 M imidazole and the purified proteins analyzed by immunoblotting with anti-SmSP2 IgG.

### Expression and purification of recombinant SmSP2 in *Pichia pastoris*

**Fig 1:**
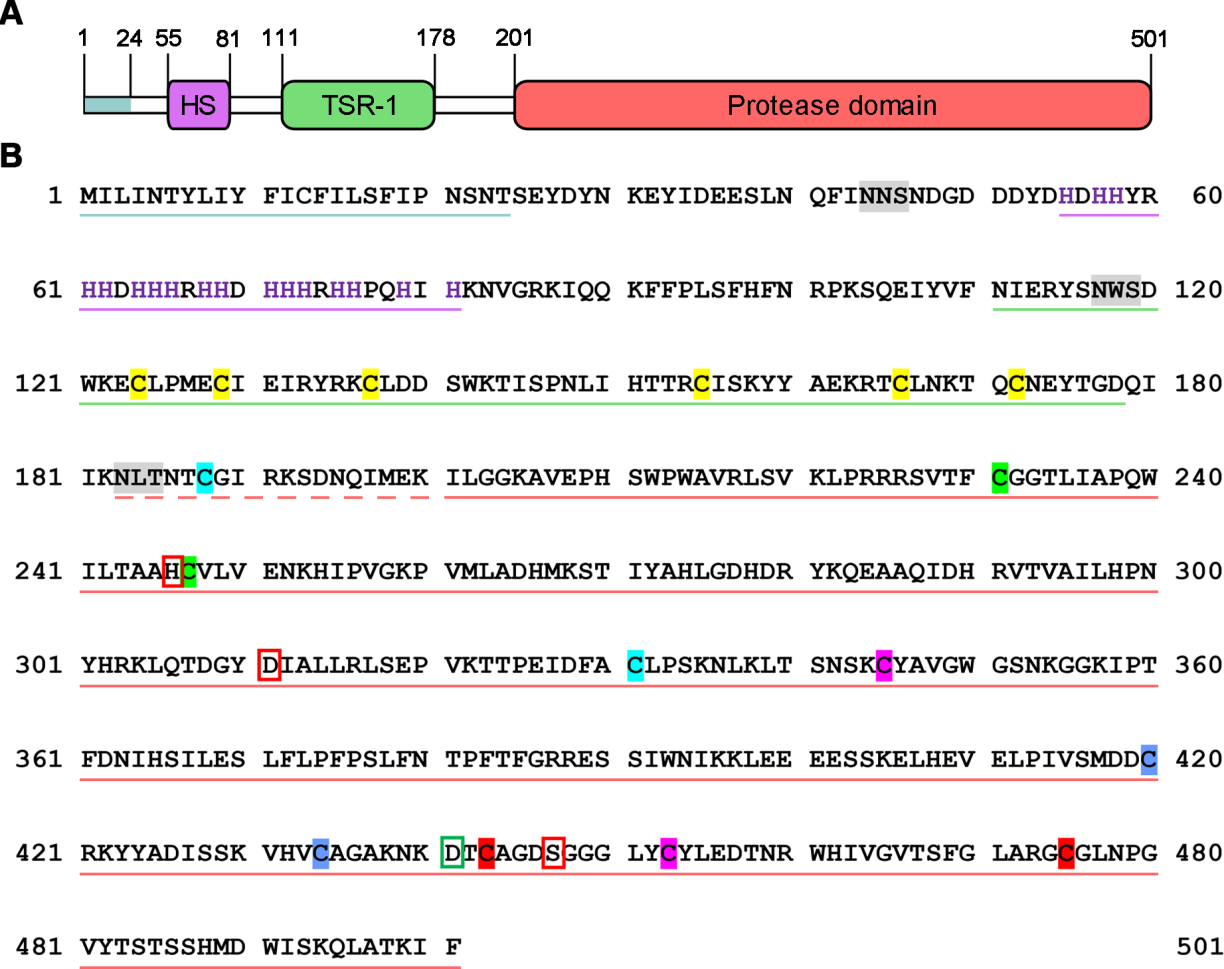
Domain organization and amino acid sequence of SmSP2. (A) Schematic diagram of the domain layout. The N-terminal signal peptide, “His stretch’ (HS), thrombospondin type 1 repeat (TSR-1) and S1 family protease domains are depicted in blue, purple, green, and red, respectively. Amino acid residue numbers are indicated. (B) The amino acid sequence of SmSP2 with the various domains color-coded by underlining as in (A). Predicted N-glycosylation sites are highlighted in grey, and His residues in the His stretch are in purple. The catalytic residues, His246, Asp311 and Ser447 are red-boxed; and Asp441 in the S1 subsite that accounts for trypsin-like activity is green-boxed. Cys residues of the protease domain that are predicted to form a disulfide are indicated by the same color; Cys residues of the TSR-1 domain are colored yellow. The propeptide is underlined with dashed red line.

The full-length SmSP2 gene (GenBank KF510120; GeneDB Smp_002150) without the N-terminal signal sequence predicted by SignalP [30] was codon-optimized for expression in *P*. *pastoris* and cloned into the pUC57 vector (GenScript) with an incorporated C-terminal His-tag (GPHHHHHH). The SmSP2 protease domain (residues 201 to 501, Fig 1) containing a short N-terminal propeptide (residues 183 to 200, Fig 1) was prepared by PCR amplification of the synthetic SmSP2 gene using the forward primer, 5´-AAGAGAGGCTGAAGC**TGCA**AACTTGACAAACACCTGTGGTATCAG-3´ that contained a Pst I restriction site, and the reverse primer, 5´-GGCCACGTGAATTCCTTAGTGATGGTGATGGTGATGAGGACC-3. Both primers contained 15 nucleotide extensions (underlined) homologous to the ends of Pst I-linearized pPICZαB vector (Thermo Fisher). The PCR product was cloned into this expression vector using the In-Fusion HD Cloning Kit according to manufacturer protocol (Clontech) and verified by DNA sequencing. Transformation of *P*. *pastoris* X-33 cells (Thermo Fisher) and protein expression were carried out as described previously [31, 32].

The yeast medium containing recombinant SmSP2 was centrifuged (3,000 *g* for 10 min), and the supernatant filtered (0.45 μm), lyophilized and dissolved in 20 mM MES buffer, pH 6.0 (to 10% of the original volume). The solution was then desalted over a Sephadex G-25 column equilibrated with the same buffer. SmSP2 was purified using chromatography on a HiTrap Benzamidine FF column (GE Healthcare Life Sciences) equilibrated with 50 mM Tris-HCl, pH 8.0, containing 0.5 M NaCl, and eluted with 50 mM glycine, pH 3.0. The pH of the eluted fractions containing SmSP2 was adjusted to 6.0 by addition of 1M Tris-HCl, pH 8.0, and enzyme fractions were stored in -80°C. The purification process was monitored with a kinetic activity assay using the fluorogenic substrate, P-F-R-AMC (AMC, 7-amino-4-methylcoumarin), and by SDS-PAGE and Western blotting.

### Expression, refolding, purification of recombinant SmSP2 in *E*. *coli*

The Champion pET directional expression kit (Thermo Fisher) was selected for bacterial expression of the SmSP2 protease domain (residues 201–501) that contained a short N-terminal propeptide (residues 183 to 200, Fig 1). The 957 bp ORF was PCR amplified from a synthetic SmSP2 gene using specific forward (5´- caccATGAATCTAACTAATACATGTGGAATACGTAAATCA-3´) and reverse (5´- AAATATTTTTGTTGCTAATTGTTTTGATATCCAA-3´) primers. The PCR product was cloned into the expression vector pET100/D-TOPO (Thermo Fisher) incorporating an N-terminal His tag and verified by DNA sequencing. Recombinant SmSP2 was produced in *E*. *coli* BL21(DE3) (Thermo Fisher), purified from inclusion bodies using Ni^2+^ chelating chromatography under denaturing conditions and the purified protein was refolded using the dialysis protocol described in [33].

### Preparation of antibodies and immunoblotting

Specific polyclonal antibodies were generated in rabbits (Moravian Biotechnology) against the bacterially-expressed SmSP2 protease domain. Antigen (50 μg in Freund’s incomplete adjuvant) was administered three times each three weeks apart. IgG was isolated from the serum by affinity chromatography with a HiTrap Protein A column (GE Healthcare Life Sciences) according to the manufacturer’s protocol. Immunoselection of SmSP2-specific IgG was carried out using a standard methodology [34]. Briefly, 800 μg of recombinant SmSP2 expressed in *E*. *coli* was resolved by SDS-PAGE and transferred to polyvinylidene difluoride membrane. Excised strips containing SmSP2 were blocked with 3% BSA in 50 mM Tris-HCl, pH 7.5, containing 150 mM NaCl and 0.1% Tween (TTBS) for 1 h, washed with TTBS, and incubated with the protein-A purified anti-SmSP2 IgG overnight at 4°C. After washing, bound antibodies were eluted with 100 mM glycine, pH 2.5 and the elution was immediately adjusted to pH 7.5 using 1 M Tris-HCl.

For immunoblotting, adult schistosome homogenate (30 μg protein) and rSmSP2 (1 μg) were resolved by SDS-PAGE (4–12% NuPAGE gel, Thermo Fisher) under reducing conditions and transferred onto a PVDF membrane. The membrane was blocked for 1 h with 3% BSA in TTBS, washed with TTBS and incubated overnight with anti-SmSP2 polyclonal IgG diluted 1:200 in TTBS. After washing with TTBS, the membrane was incubated for 1 h with goat horseradish peroxidase-conjugated anti-rabbit IgG (Sigma-Aldrich) at a dilution of 1:10,000. After washing with TTBS, the membrane was developed with Luminata Crescendo Western HRP substrate (Merck) and imaged using an ImageQuant LAS 4000 biomolecular imager (GE Healthcare Life Sciences).

### Immunofluorescence microscopy

Adult *S*. *mansoni* males and females were carefully separated over ice, washed three times in 100 mM sodium phosphate, pH 7.4, containing 154 mM NaCl (PBS), fixed in Bouin’s solution (Sigma-Aldrich) for 45 min at 25°C, and embedded in paraffin blocks. Sections (6–8 μm each) were deparaffinized in xylol, rehydrated in an ethanol series of decreasing ethanol concentration and boiled in a water bath in 10 mM sodium citrate, pH 6.0, for 15 min. Cooled sections were rinsed with PBS, permeabilized in PBS containing 0.25% Triton X-100 (PBS/Triton) for 20 min, and blocked overnight at 4°C with 2% BSA in PBS/Triton. After washing in PBS/Triton, the sections were incubated for 60 min at 4°C with rabbit polyclonal anti-SmSP2 IgG diluted 1:25 in PBS/Triton containing 1% BSA. Sections were then washed three times in PBS/Triton and incubated for 30 min at 25°C with anti-rabbit IgG Alexa 647-conjugated secondary antibody (Thermo Fisher) diluted 1:500 in PBS/Triton. After washing with PBS/Triton, the sections were mounted in Prolong Diamond antifade reagent containing DAPI (Thermo Fischer). The fluorescence was visualized using an Olympus IX83 microscope equipped with PCO edge 5.5 camera and CoolLED pE-4000 LED illumination system. DAPI signal was detected using excitation diode 365 nm and emission filter 440/40 nm, Alexa 647 using diode 635 nm and emission filter 700/75. Appropriate lightning settings were determined using control slides probed with pre-immune serum to define the background signal threshold. Image stacks of optical sections were processed using the Fiji software.

### Active site labeling of SmSP2

Recombinant SmSP2 expressed in *P*. *pastoris* (1 μg) was incubated 1 h at 37°C with 1 μM activity-based probe BoRC [35] in 50 mM Tris-HCl, pH 8.0. The competitive labeling reaction was also performed after treatment of SmSP2 for 15 min at 37°C with 1 mM serine protease inhibitor Pefabloc SC (Sigma-Aldrich). The labeled SmSP2 was precipitated with acetone, resolved by SDS-PAGE, and visualized in-gel using a Typhoon 9410 imager (GE Healthcare) as described previously [36].

### Kinetic SmSP2 activity and inhibition assays

SmSP2 activity was measured in continuous kinetic assays using the following peptidyl fluorogenic AMC substrates (Bachem): Z-F-R-AMC (Z, Benzyloxycarbonyl), Bz-F-V-R-AMC (Bz, Benzoyl), P-F-R-AMC, Boc-L-R-R-AMC (Boc, t-Butyloxycarbonyl), Boc-Q-A-R-AMC, Boc-V-L-K-AMC, Suc-A-A-F-AMC (Suc, Succinyl), Suc-A-A-P-F-AMC, MeOSuc-A-A-P-V-AMC (MeOSuc, 3-Methoxysuccinyl), Z-G-G-L-AMC, Z-V-K-M-AMC, Boc-L-G-R-AMC, Z-V-V-R-AMC, V-P-R-AMC, Z-R-R-AMC, R-R-AMC, R-AMC and Boc-L-L-V-Y-AMC. Assays were performed at 37°C in white 96-well microplates in a total volume of 100 μl. Recombinant SmSP2 expressed in *P*. *pastoris* (10 ng) was pre-incubated for 10 min at 37°C in 80 μl of 0.1 M Tris-HCl, pH 8.0. The reaction was initiated by adding 20 μl of substrate solution (50 μM final concentration) in the same buffer. Proteolytic activity was measured continuously in an Infinite M1000 microplate reader (Tecan) at excitation and emission wavelengths of 360 and 465 nm, respectively. The pH activity profile was determined in 50 mM citrate, 100mM phosphate (pH range 3.0–7.5), and 0.1M glycine (pH range 8.0–11.0) using the P-F-R-AMC substrate. The pH profile of SmSP2 activity in schistosome homogenates (1–5 μg of protein) was measured analogously, but in the presence of 10 μM E-64 and 1 mM EDTA to prevent undesired proteolysis by cysteine proteases and metalloproteases, respectively. Protease activity was expressed as the remaining portion that was sensitive to 1 mM Pefabloc SC. For inhibition measurements, inhibitors were added to the 80 μL pre-incubation solution at the final concentrations listed in Table 1. After 10 min at 37°C, reactions were initiated by the addition of substrate. For pH stability determinations, SmSP2 was incubated in 50 mM citrate, 100mM phosphate (pH range from 3.0 to 7.0) or 0.1M glycine (pH range from 8.0 to 11.0). After 1, 4 and 20 h, aliquots containing 10 ng SmSP2 were taken and SmSP2 activity was measured using P-F-R-AMC as described above. All measurements were done in triplicate.

**Table 1:** Sensitivity of rSmSP2 to protease inhibitors.

Inhibitor^a^	Target protease^b^	Concentration (μM)	Inhibition (%)^c^
**Pefabloc SC**	SP	1000	100
**Benzamidine**	SP	1000	100
**3,4-dichlorcoumarin**	SP	100	71.4 ± 3.3
**Antipain**	SP, CP	20	100
**Leupeptin**	SP, CP	20	100
**BPTI (Aprotinin)**	SP	10	87.0 ± 2.6
**STI**	SP	10	91.8 ± 0.9
**α-1-antichymotrypsin**	SP	1	13.2 ± 2.4
**α-1-antitrypsin**	SP	1	79.0 ± 0.9
**Antithrombin III**	SP	1	99.1 ± 0.3
**PAI-1**	SP	1	100
**E-64**	CP	10	7.2 ± 0.9
**Pepstatin A**	AP	1	0
**EDTA**	MP	1000	3.2 ± 0.9
**Bestatin**	MP	1	4.5 ± 2.0
**Phenanthroline**	MP	1000	26.9 ± 4.7
**α-2-macroglobulin**	all types	0.1	61.6 ± 0.4

^a^ Abbreviations: BPTI (bovine pancreatic trypsin inhibitor), STI (soybean trypsin inhibitor), E64 (trans-epoxysuccinyl-L-leucylamido(4-guanidino)butane), PAI-1 (plasminogen activator inhibitor-1).

^b^ The target proteases are classified based on catalytic type into aspartic (AP), cysteine (CP), serine (SP) proteases and metalloproteases (MP).

^c^ rSmSP2 was pre-incubated with the given inhibitor and remaining activity was measured in a kinetic assay with the fluorogenic substrate P-F-R-AMC. The mean values ± S.D. of three replicates are expressed as the percentage inhibition relative to uninhibited controls.

### Interaction of SmSP2 with protein substrates

Recombinant SmSP2 expressed in *P*. *pastoris* (300 ng) was incubated at 37°C with 1–20 μg of human plasminogen (hPLG, R&D Systems), high molecular weight human plasma kininogen (HMWK; Merck), human tissue plasminogen activator (tPA), human serum albumin (HSA), human hemoglobin, calf collagen type I, human fibronectin and rabbit immunoglobulin G (all Sigma-Aldrich) in 25 μL 100 mM Tris-HCl, pH 8.0. After incubation (between 1 and 48 h), the reaction was stopped by adding Pefabloc SC (final concentration 1 mM) and 20 μl of the reaction was resolved by SDS-PAGE (4-12% Nupage gel) and stained with Coomassie Brilliant Blue G250. In control experiments, protein was incubated in the absence of SmSP2 and analyzed under identical conditions. Processing products generated during HMWK degradation were identified by mass spectrometry, the reaction mixture was analyzed using LC-MS/MS as described previously [32, 37]. To analyze the activation of hPLG and tPA by SmSP2, aliquots of the reaction mixtures containing 100 ng of hPLG or tPA were withdrawn at different time intervals and activity was measured in a kinetic assay using 50 μM Boc-V-L-K-AMC or Z-G-G-R-AMC, respectively, in 100 mM Tris-HCl, pH 7.5, containing 100 mM NaCl. Proteolytic activity was measured continuously as described above. In control experiments, SmSP2, hPLG or tPA alone were analyzed under identical conditions.

### Hydrolysis of peptide hormones by SmSP2

Extended bradykinin (Ac-SLMKRPPGFSPFRSSR-amide, Ac, acetyl) was synthesized as a peptidyl amide by Fmoc solid phase chemistry in an ABI 433A peptide synthesizer (Applied Biosystems), as described previously [19, 38]. Recombinant SmSP2 expressed in *P*. *pastoris* (200 ng) was incubated at 37°C for 1 to 16 h with 25 nmol of bradykinin, lysyl-bradikinin (kallidin), vasopresin (all Sigma-Aldrich) or extended bradykinin in 0.1 M Tris-HCl, pH 8.0, in a total volume of 50 μL. The reaction was stopped by adding TFA to a final concentration of 1%. The resulting fragments were purified by reverse-phase HPLC using a Luna C18 column (25 x 0.46 cm, Phenomenex) and the TFA/acetonitrile system, and characterized by MS/MS [32, 37].

### Hydrolysis of peptides by cultured schistosomes

Adult worms were washed and treated as described above (section Preparation of schistosome extract, excretory/secretory products collection and purification of native SmSP2). Five adult schistosome pairs were then placed into clear, 24-well, flat-bottom plates (Costar) containing 500 μL Basch Medium 199 [28], supplemented with 100 units/mL penicillin and 100 μg/mL streptomycin (without fetal bovine serum). Human vasopressin or extended bradykinin (10 μM) was added and the incubation continued for 16 h at 37°C under a 5% CO2 atmosphere. In control experiments, the peptides were incubated in the same system but in the absence of schistosomes. After incubation, the samples were ZipTiped and the resulting fragments were analyzed using MALDI-TOF performed on an UltrafleXtreme (Bruker Daltonik) operated in reflectron mode with an acceleration voltage of 25 kV and a pulsed ion extraction of 120 ns. Desorption and ionization were achieved using a Smartbeam II laser. α-Cyano-4-hydroxycinnamic acid was used as a matrix. The data were acquired from m/z 220 to 3700 and analyzed with the FlexAnalysis 3.3 software (Bruker Daltonik). The mass spectra were externally calibrated using a Peptide Calibration Standard I (Bruker Daltonik) and averaged from 3000 laser shots.

### Subsite profiling of SmSP2 by a positional scanning synthetic combinatorial library (PS-SCL) and by multiplex cleavage assays

Synthesis of the PS-SCL has been previously described [39]. The assays were carried out in black 96-well microplates in 0.1 M Tris-HCl, pH 8.0, containing 0.01% Tween 20 and initiated by addition of recombinant SmSP2 (10 ng). Release of 7-amino-4-carbamoylmethylcoumarin (ACC) was measured in an SpectraMax Gemini fluorescence spectrometer (Molecular Devices) with excitation and emission wavelengths set to 380 and 460 nm, respectively.

The Multiplex Substrate Profiling by Mass Spectrometry (MSP-MS) assay was performed as previously described [29]. SmSP2, human plasma kallikrein (Sigma-Aldrich) and bovine trypsin Sigma-Aldrich) (all 17 nM) were incubated in triplicate with a mixture of 228 synthetic tetradecapeptides (0.5 uM each) in 10 mM Tris-HCl, pH 8.0. After 15, 60, and 240 minutes, 20 μL aliquots were removed, quenched with 6.4 M guanidinium chloride, immediately frozen at -80°C. Control reactions were performed by treating enzymes with guanidinium chloride prior to peptide exposure. Samples were acidified to < pH 3.0 with 1% formic acid, desalted with C18 LTS tips (Rainin), and injected into a Q Exactive Mass Spectrometer (Thermo) equipped with an Ultimate 3000 HPLC (Thermo). Peptides separated by reverse phase chromatography on a C18 column (1.7 um bead size, 75 um x 25 cm, heated to 65°C) at a flow rate of 300 nL min^-1^ using a 55-min. linear gradient from 5% B to 30% B, with solvent A: 0.1% formic acid in water and solvent B: 0.1% formic acid in acetonitrile. Survey scans were recorded over a 150–2000 m/z range at 70000 resolutions (AGC target 1×106, 75 ms maximum). MS/MS was performed in data-dependent acquisition mode with HCD fragmentation (30 normalized collision energy) on the 10 most intense precursor ions at 17500 resolutions (AGC target 5×104, 120 ms maximum, dynamic exclusion 15 s).

Peak integration and peptide identification were performed using Peaks software (Bioinformatics Solutions Inc.). Quantification data are normalized by LOWESS and filtered by 0.3 peptide quality. Missing and zero values are imputed with random numbers in the range of the average of smallest 5% of the data ± sd. Differences between each time point and no-enzyme control were analyzed for statistical significance by multiple t-test. When compared to the control reaction, peptide cleavage products with >10-fold change in peak area intensity and p-value <0.05 were identified and the peptide sequence corresponding to the P4 to P4' subsite positions were used to make IceLogo frequency plots [40]. Mass spectrometry data and results can be accessed here: ftp://massive.ucsd.edu/MSV000081747.

### Molecular modeling of SmSP2

A spatial model of the SmSP2 protease domain was constructed by homology modeling, as described previously [37]. Briefly, the X-ray structures of human mannan-binding lectin serine protease 1 (MASP-1) and bovine trypsin ((Protein Data Bank (PDB) entries: 3GOV and 1JRT, respectively) were used as templates. The homology module generated by the MOE program (Chemical Computing Group, Canada) was used to model the SmSP2 structure. The inhibitor conformation was refined by applying the LigX module of the MOE and the final binding mode of the inhibitor was selected by the best-fit model based on the London dG scoring function and the generalized Born method [37]. Molecular images were generated with UCSF Chimera (http://www.cgl.ucsf.edu/chimera/).

## Results

### SmSP2 is a S1 family protease with a unique domain organization

The SmSP2 open reading frame consists of 1,506 bp that encode a protein of 501 amino acid residues with a theoretical molecular mass of 58 kDa. The predicted signal sequence is 24 residues long and the amino acid sequence contains three potential N-glycosylation sites (Fig 1). Based on sequence homology analysis, we describe SmSP2 as a multi-domain protein made up of an N-terminal region (residues 25 to 110), a thrombospondin-1 type 1 repeat (TSR-1) domain (residues 111 to 178) and a S1 family serine protease domain at the C-terminus (residues 201 to 501). The N-terminal region lacks significant similarity with other published protein sequences in databases. The striking feature of this region is the presence of a stretch of histidine residues (the ‘His stretch’—residues 55 to 81) which suggests that SmSP2 may bind metal ions.

The TSR-1 domain has been identified in multiple protein families and occurs in more than 40 human proteins, *e*. *g*., thrombospondins, ADAMTS (A Disintegrin And Metalloproteinase with Thrombospondin Motif), properdin and some complement factors [41]. It is known to mediate cell adhesion, protein-protein interactions, glycosaminoglycan binding and inhibit angiogenesis [42, 43]. The TSR-1 of SmSP2 consists of about 60 residues and its sequence contains all of the important conserved features of TSR-1 domains (S1 Fig): a cysteine residue pattern and conservation of tryptophan and arginine residues forming so called W and R layers [44].

The catalytic protease domain of SmSP2 belongs to the S1 family of serine proteases and has about 30% identity with other members of this family (S2 Fig). The protease domain of SmSP2 possesses a catalytic triad of His246, Asp311 and Ser447 (corresponding to amino acid residues 57, 102 and 195 by standard chymotrypsinogen numbering), which is typical of S1 family proteases. In addition, the amino acids surrounding the catalytic-triad residues have the highest sequence identity with other S1 family enzymes (S2 Fig). Cysteine residues in the catalytic domain form four conserved disulfide bonds that can be predicted by the alignment with the solved crystal structures of S1 family proteases (S2 Fig). Moreover, an additional cysteine residue, Cys311, is likely to form a disulfide bond with a Cys188 in the N-terminal region. An analogous disulfide bond is described, for example, in bovine chymotrypsinogen, human plasmin, urokinase (uPA), tissue plasminogen activators (tPA) and MASP-1. In bovine trypsin, Asp189 is located at the bottom of the S1 binding site and determines the trypsin-like specificity for substrates with Arg/Lys in the P1 position [45]. This residue is conserved in the sequence of SmSP2 (Asp441). When compared to bovine chymotrypsin, the S1 family holotype protease [46], SmSP2 has three insertions (S2 Fig) located between residues 222 and 226 (insertion-222), 251 and 268 (insertion-251), and between residues 358 and 400 (insertion-358). Whereas protein sequences corresponding to the short insertions-222 and -251 are found in uPA/tPA and MASP1, respectively, the long insertion-358 is unique to SmSP2.

SmSP2 orthologs are found in: (i) other schistosome species—*S*. *japonicum* (GenBank: AAW24683.1) and *S*. *haematobium* (XP_012796372.1) sharing 80% and 78% sequence identity, respectively; (ii) other trematodes, including, *Fasciola hepatica* (identified in the transcriptome database [47] - 53% identity), *Opisthorchis viverrini* (XP_009167273.1–49% identity), *Clonorchis sinensis* (GAA32831.2–47% identity); and (iii) the sequences of cestodes such as *Hymenolepis microstoma* (CDS25513.1), *Echinococcus multilocularis* (CDI97096.1), *Echinococcus granulosus* (CDI97096.1), and *Taenia solium* (ADP89566.1) sharing 27–39% identities (S3 Fig). All of the ortholog sequences contain the TSR-1 and protease domains, however, they differ in the N-terminal region in length and the presence of the His stretch that is unique to trematodes. Moreover, the insertion-358 in the cestode sequences is shorter and contains two additional Cys residues that might potentially form a disulfide stabilizing this structure (S3 Fig). To conclude, SmSP2 and its orthologs are serine proteases with unique domain organization found exclusively in the phylum Platyhelminthes.

## Homology model of SmSP2 reveals the trypsin-like active site pocket shielded by additional loops

**Fig 2:**
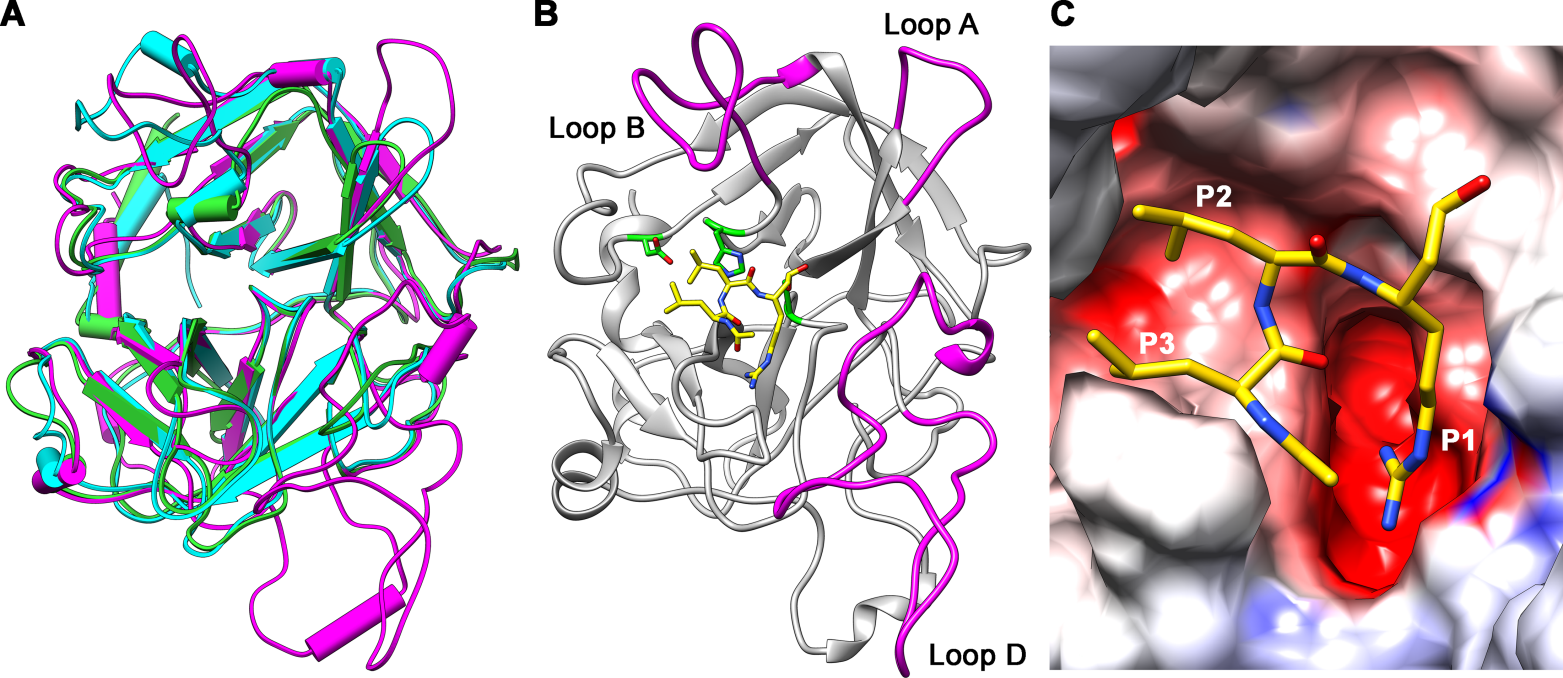
Homology model of the SmSP2 protease domain. The model was built using as a template the X-ray structures of bovine trypsin (PDB 1JRT) and human MASP-1 (PDB 3GOV). (A) A superposition of the SmSP2 model (magenta), the bovine trypsin (green) and MASP-1 (cyan) crystal structures in a cylinder representation. (B) A view from the top on the SmSP2 active site with covalently bound substrate-like inhibitor leupeptin (N-acetyl-L-leucyl-L-leucyl-L-argininal). Carbon atoms of leupeptin are yellow; heteroatoms have the standard color coding (N, blue; O, red). SmSP2 catalytic residues are green. The active site is partially blocked by loops A, B and D (magenta) that are formed by insertions in SmSP2 sequence compared to trypsin sequence. (C) A surface representation of the SmSP2 active site colored by electrostatic potential (at a scale from -10 kT/e (red) to +10 kT/e (blue)). Inhibitor leupeptin is colored as in (B).

A spatial model of the SmSP2 protease domain was constructed by homology modelling to provide a structural framework to analyze structure-activity relationships. The X-ray structure of bovine trypsin (PDB code 1JRT) and human MASP-1 (PDB code 3GOV) were used as templates. Fig 2A shows that the SmSP2 protease domain has the conserved architecture of S1 family proteases which consists of two six-stranded β-barrel domains packed against each other. The catalytic amino acid residues, His246, Asp311 and Ser447 are located at the junction between these β-barrel domains. The major sequence insertions in SmSP2 compared to trypsin are located at surface-exposed loops surrounding the substrate binding region (Fig 2B). SmSP2 insertions-222 and -251 are located on the top of the loops B and A, respectively (Perona and Craik nomenclature [48]). Their structural analogs can be found in the structure of the MASP-1 catalytic domain where they putatively interact with substrates [49]. The insertion-358 containing 43 residues are located at loop D; however, it has no structural analog in the PDB database (www.rcsb.org) to serve as a template for modeling. The secondary structure prediction did not reveal any conformational element in the loop, suggesting an unstructured character and flexibility that might be involved in interactions during substrate binding.

The ligand binding mode of the SmSP2 protease domain was further analyzed using leupeptin (N-acetyl-L-leucyl-L-leucyl-L-arginal), a transition-state analog protease inhibitor that inhibits SmSP2 (Table 1). Leupeptin was docked into the SmSP2 active site based on the crystallographic complex of this inhibitor with trypsin (PDB code 1JRT). The docking model (Fig 2) suggests that the arginal residue of the inhibitor forms a covalent hemi-acetal linkage with the catalytic Ser447 whereas leupeptin’s side chain binds to a deep negatively charged S1 subsite pocket containing Asp441 at the bottom. This type of S1 binding subsite is the primary substrate specificity determinant of trypsin-type proteases and responsible for a substrate/inhibitor preference for basic residues at the P1 position [45].

To conclude, the model of the SmSP2 protease domain indicates that it is a S1 family protease with an trypsin-like substrate binding groove; shielded by surface exposed loops that surround the active site, namely, insertions-222 and -358, may modulate SmSP2’s selectivity.

### Recombinant expression of SmSP2 and identification of native SmSP2

**Fig 3:**
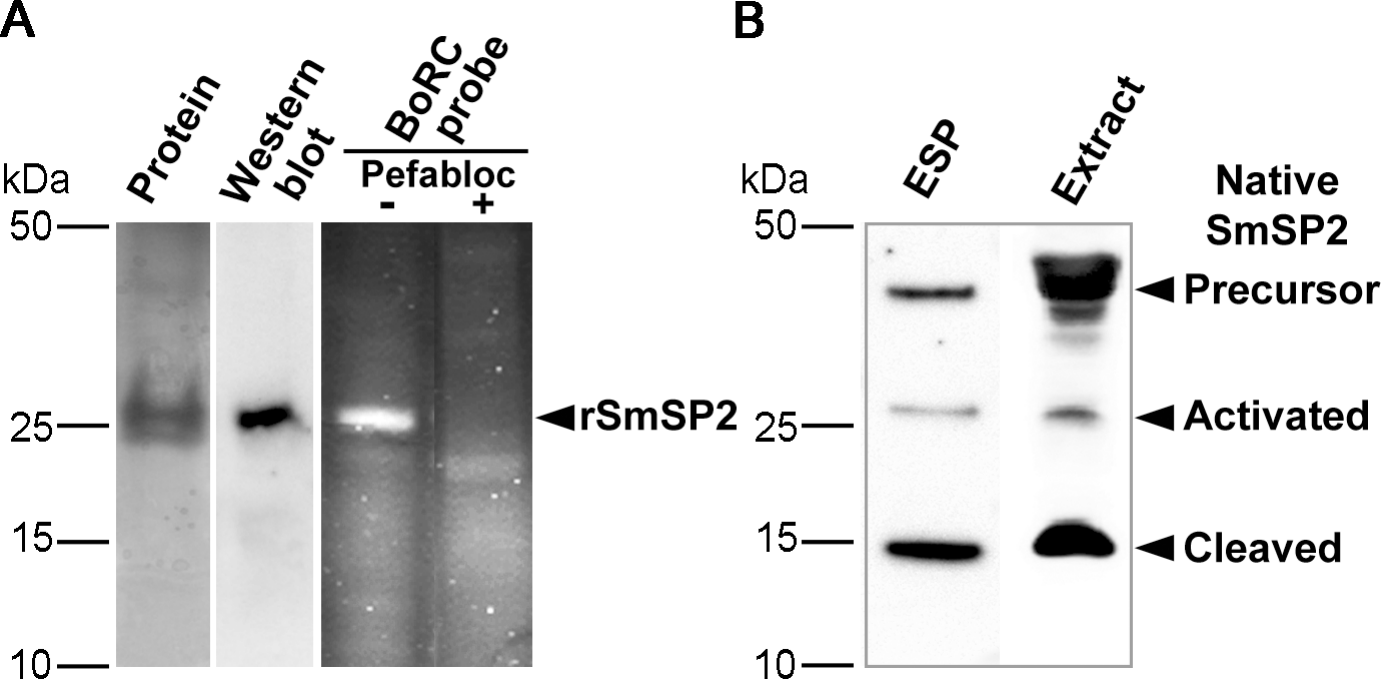
Preparation of recombinant SmSP2 and identification of native SmSP2. (A) The recombinant protease domain of SmSP2 (rSmSP2) expressed in *P*. *pastoris* was resolved by SDS-PAGE and protein-stained or visualized by polyclonal anti-rSmSP2 IgG. For in-gel activity-based labeling, rSmSP2 was incubated with the fluorescent active site probe, BoRC, resolved by SDS-PAGE and visualized using a fluorescence scanner. The competitive labeling was performed with the serine protease inhibitor, Pefabloc SC. (B) Protein extracts of *S*. *mansoni* adult worms and their ESP were resolved by SDS-PAGE and visualized by the anti-rSmSP2 IgG.

The protease domain of SmSP2 (rSmSP2, residues 183–501) was expressed in *P*. *pastoris* and purified. The active enzyme cleaved Z-F-R-AMC and migrated on SDS-PAGE as a single band of approximately 28 kDa (Fig 3A), consistent with the expected molecular mass of 30 kDa. rSmSP2 was visualized using the activity-based probe Bodipy-F-P-R-Cmk (BoRC) (Fig 3A) [35]; labeling by the probe was prevented by pre-incubation of the enzyme with the serine protease inhibitor, Pefabloc SC. Rabbit polyclonal antibodies, raised against the SmSP2 protease domain expressed in *E*. *coli*, reacted with the rSmSP2 by immunoblotting (Fig 3A). In homogenates of adult schistosomes, the antibody recognized three bands of approximately 45, 28 and 15 kDa (Fig 3B), which correspond to the predicted mass of the SmSP2 precursor, the activated SmSP2 protease domain without N-terminal domains and the two-chain form derived by further processing, respectively. Based on its molecular size, we hypothesize that this form was clipped in the insertion-358 loop D. Analogous immunoreactive bands were also demonstrated in the ESP of adult schistosomes (Fig 3B), indicating that SmSP2 is released into the host environment.

The full length SmSP2 sequence contains a stretch of histidine residues that may interact with metal ions. Indeed, native SmSP2 from schistosome extracts bound to a Ni^2+^- affinity chromatography column and eluted using imidazole in solution (S4 Fig). The eluate contained the three forms of SmSP2 described above. The data also indicate that the activated and clipped protease domains (28 and 15 kDa bands) are disulfide-linked to the N-terminal portion of the molecule that contains the histidine stretch.

## Substrate and inhibitor specificity classifies SmSP2 as a trypsin-like enzyme

**Fig 4:**
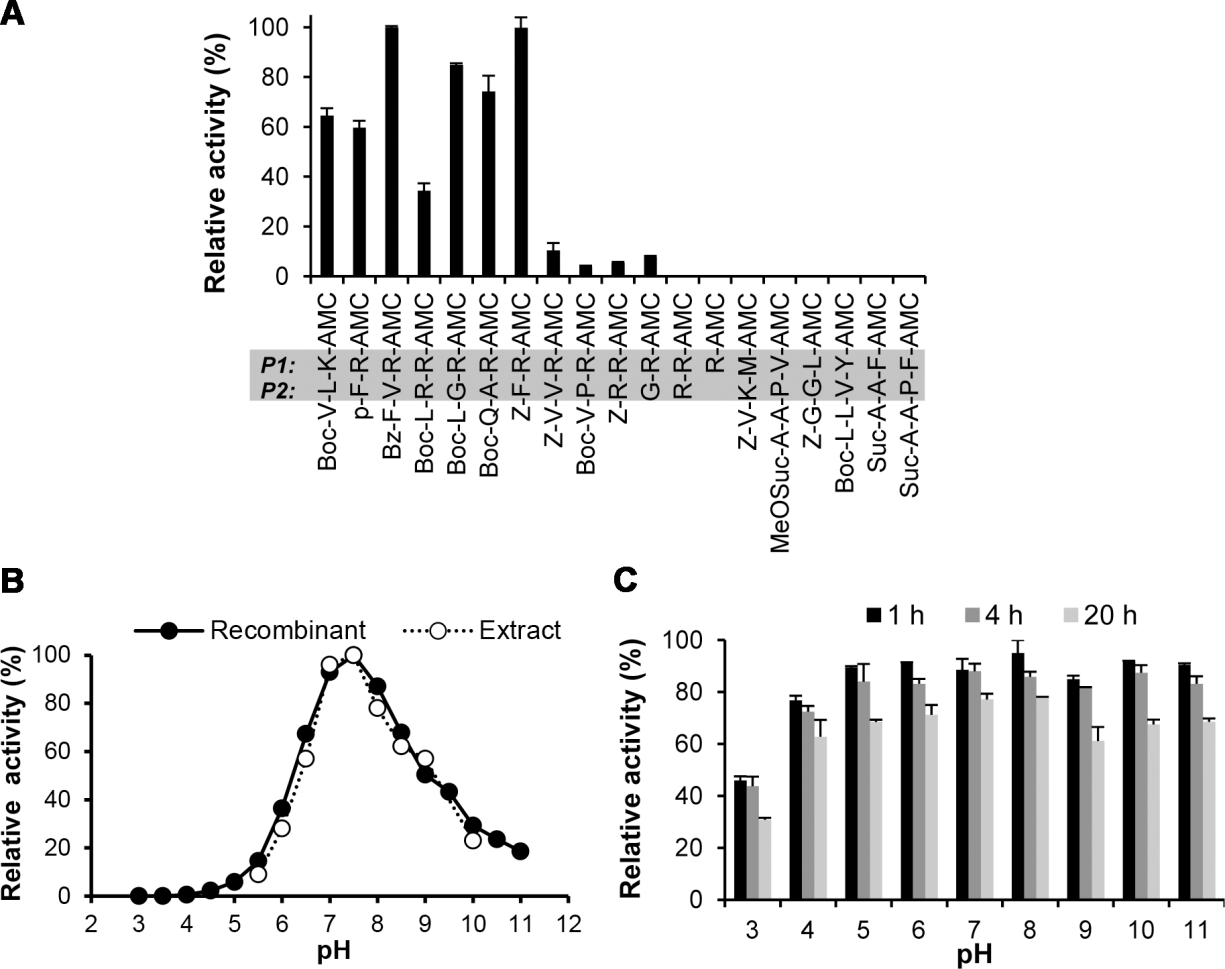
Substrate specificity and pH profile of SmSP2. (A) Activity of rSmSP2 was probed using a panel of peptidyl fluorogenic substrates used to assay trypsin-like and chymotrypsin/elastase-like serine proteases. Substrate hydrolysis was measured in a kinetic assay at pH 8.0. The mean values ± S.D. of three replicates are normalized to the maximum value. Amino acid residues at P1 and P2 positions are highlighted by the grey bar. (B) The pH profiles of rSmSP2 and native SmSP2 activity in extracts of adult worms. Activity was measured in a kinetic assay using the fluorogenic substrate P-F-R-AMC. The native activity (sensitive to the serine protease inhibitor Pefabloc SC) was measured in the presence of 10 μM E-64 and 1 mM EDTA to prevent undesired proteolysis of the substrate by cysteine proteases and metalloproteases, respectively. The mean values of three replicates, expressed as a percentage normalized to the highest value, are shown (standard deviation values are within 5% of the mean). (C) The pH stability of rSmSP2. Activity of rSmSP2 was measured at pH 8.0 in a kinetic assay as in (B) after incubation of the enzyme at pH 3 to 11 for different times. The mean values of three replicates, expressed as a percentage normalized to activity of non-incubated rSmSP2, are shown.

The substrate specificity of rSmSP2 expressed in *P*. *pastoris* was initially explored using a panel of specific peptidyl fluorogenic substrates. Two sets of diagnostic protease substrates were used: (i) substrates with a basic amino acid residue (Arg and Lys) at the P1 position that are cleaved by trypsin-like serine proteases, and (ii) substrates containing bulky hydrophobic (Phe and Tyr) or aliphatic residues (Val, Leu and Met) at P1 that are cleaved by chymotrypsin- or elastase-like serine proteases, respectively [50]. The data show that rSmSP2 predominantly hydrolyzed trypsin substrates (Fig 4A). Activity was greatest with Bz-F-V-R-AMC and Z-R-R-AMC, whereas less activity was measured with Z-V-V-R-AMC, Z-V-P-R-AMC and Z-R-R-AMC. The cleavage of related short substrates with free N-termini occurs only very slowly (G-R-AMC) or not at all (R-R-AMC and R-AMC), suggesting that SmSP2 is not an aminopeptidase trimming N-terminal residues of substrates. The chymotrypsin/elastase substrates were not effectively hydrolyzed.

The pH activity profile of rSmSP2 was determined using the P-F-R-AMC and was similar to that of the serine protease activity in the schistosome adult homogenate (Fig 4B). Effective hydrolysis was observed between pH 6.0 and 10.0 with optimal activity around pH 8.0. No SmSP2 activity was detected below pH 5.0, although rSmSP2 is stable above pH 4.0 (Fig 4C).

**Fig 5:**
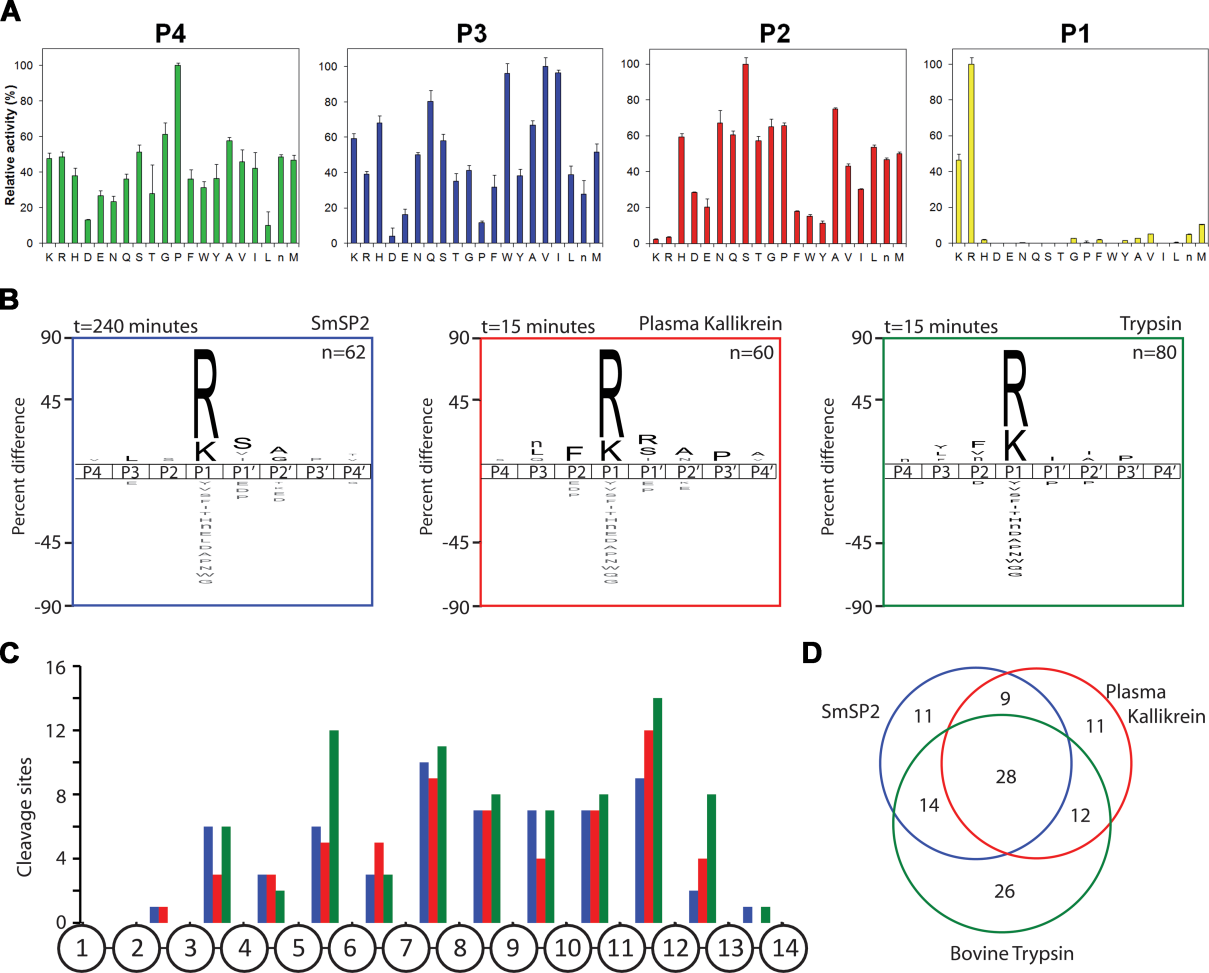
Substrate specificity of rSmSP2. (A) The P1 to P4 specificity of rSmSP2 was determined by a positional scanning-synthetic combinatorial library. The X-axis indicates 20 amino acids held constant at each position (n is norleucine). The Y-axis represents activity related to the most preferred amino acid (100%). (B) The P4 to P4′ specificity profiles of rSmSP2, human plasma kallikrein, and bovine trypsin were obtained using a multiplex combinatorial library (MSP-MS). The iceLogo substrate profiles were generated from the pattern of cleavage events after incubation with the 14-mer library. Amino acids that are most frequently found at each position are shown above the horizontal line, whereas amino acids that less frequently observed are below. (C) Spatial distribution of cleavage sites within the 14-mer peptide scaffold. (D) The Venn diagram shows the number of unique and shared cleavage sites.

To detail the cleavage specificity of rSmSP2, two distinct methods for unbiased substrate profiling were employed. First, a positional scanning-synthetic combinatorial library (PS-SCL) [39] was used to analyze specificity at the substrate positions P1 to P4 (Fig 5A). The cleavage map shows that rSmSP2 prefers basic residues (Lys and Arg) at the P1 position. The P2 and P3 positions display promiscuous specificity, although basic residues at the P2 position, and acidic residues and Pro at the P3 position are unfavorable. Some degree of selectivity was measured for Pro at P4. Second, a sensitive multiplex substrate profiling by mass spectrometry (MSP-MS) was performed with a library of 228 tetradecapeptides [51] to analyze the subsites on both sides of the scissile peptide bond (Fig 5B). The MSP-MS profile confirmed the strong preference for Arg and Lys residues at the P1 position. At the primed sites, the preferred residues were Ser at the P1′, Ala and Gly at P2′ positions, respectively. Compared to human plasma kallikrein and bovine trypsin, the MSP-MS cleavage profile of SmSP2 were similar (Fig 5D) with 28 identical cleavage sites. However, trypsin produced 26 unique cleavages compared to 11 cleavages unique for rSmSP2 or plasma kallikrein. In general, cleaved peptide bonds occurred away from the amino and carboxyl termini confirming endopeptidase mode of action of all three analyzed enzymes (Fig 5C).

The inhibitor specificity of rSmSP2 was investigated using a panel of small molecule and protein inhibitors (Table 1). rSmSP2 was highly sensitive to inhibitors of S1 family serine proteases, including the small-molecules, Pefabloc SC, benzamidine, leupeptin, antipain, and 3,4-dichlorcoumarin, and the proteinaceous soybean trypsin (STI) and bovine pancreatic trypsin inhibitors (BPTI). rSmSP2 was not affected by inhibitors of aspartic, cysteine and metallo-proteases. Protein inhibitors of the serpin family inhibited rSmSP2 with variable efficiencies: PAI-I, α-1-antitrypsin and anti-thrombin III, which interact with trypsin-like proteases, inhibited rSmSP2 activity strongly, whereas the chymotrypsin protease inhibitor, α-1-antichymotrypsin, was weakly effective.

To summarize, SmSP2 hydrolyzes substrates as an endopeptidase and has a strict specificity for basic residues at P1.

## SmSP2 releases bradykinin from kininogen and activates plasmin

**Fig 6:**
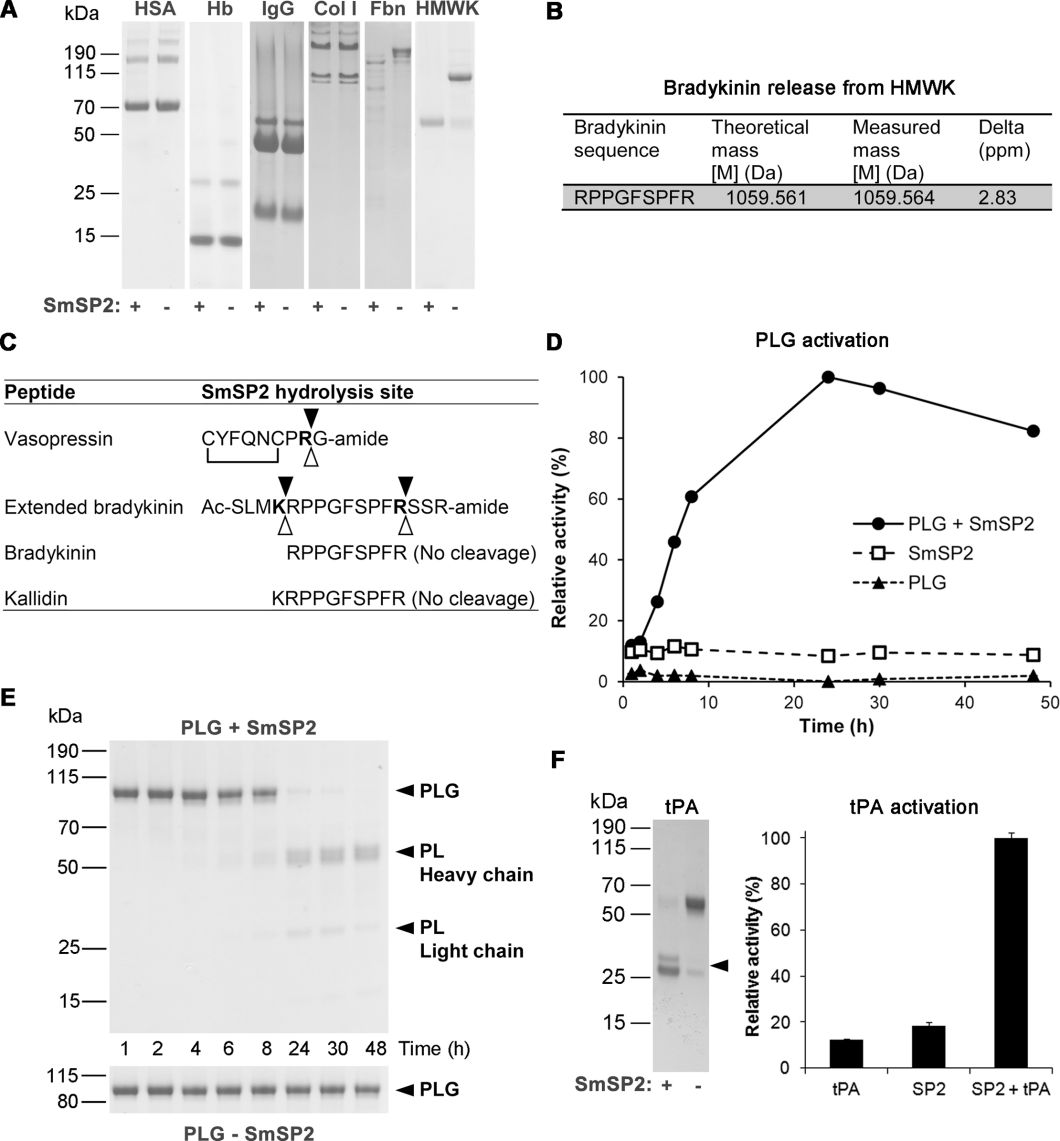
Processing of host-derived proteins and peptides by rSmSP2. (A) Human serum albumin (HSA), hemoglobin (Hb), immunoglobulin G (IgG), collagen type I (Col I), fibronectin (Fbn) and high molecular weight kininogen (HMWK) were incubated for 16 h at pH 8.0 in the presence (+) or absence (-) of rSmSP2. The reaction mixtures were subjected to SDS-PAGE and protein stained. (B) HMWK was incubated with rSmSP2 and the reaction mixture was subjected to LC-MS/MS analysis to identify bradykinin peptide released from HMWK. (C) Peptide hormones were incubated with rSmSP2 or with live adults maintained in culture and the cleavage positions (full triangles for rSmSP2, open triangles for adult schistosomes) were identified by mass spectrometry. Residues at the P1 position are in bold and the disulfide connectivity of vasopressin is indicated. (D) Human plasminogen (PLG) was incubated in the presence or absence of rSmSP2 and the reaction mixture was analyzed at different time points. Plasmin proteolytic activity generated during plasminogen processing by rSmSP2 was determined in a kinetic assay with Boc-V-L-K-AMC. Mean values of triplicates are expressed relative to the maximum value (100%). The S.D. values of three replicates are within 10% of the mean. All experiments were performed at least twice with similar results. (E) The processed forms were resolved by SDS-PAGE and visualized by protein staining. The positions for PLG, and plasmin (PL) heavy and light chains are indicated. (F) Human tissue plasminogen activator (tPA) was incubated for 16 h at pH 8.0 in the presence/absence of rSmSP2 and analyzed by SDS-PAGE with protein staining; proteolytic activity generated during tPA processing was monitored in a kinetic assay using Z-G-G-R-AMC. Mean values ± s.d. of triplicates are expressed relative to the maximum value (100%). Two chain tPA is indicated with an arrow.

The activity of rSmSP2 towards host-derived macromolecular substrates was tested with several human and bovine proteins. After incubation, the mixtures were subsequently analyzed by SDS-PAGE (Fig 6A). No hydrolysis was observed for hemoglobin and serum albumin, the two major protein components of vertebrate host blood. Also, neither immunoglobulin G nor collagen was cleaved by rSmSP2. On the other hand, rSmSP2 completely hydrolyzed the blood clotting protein, fibronectin. We also analyzed the processing of three blood proteins: HMWK, tPA and human plasminogen.

Amino acid sequencing showed that HMWK was processed to the kininogen light chain (Fig 6A), and that the heavy chain was completely fragmented. LC-MS/MS analysis of the reaction mixture revealed the release of the HMWK-derived vasodilatory nonapeptide, bradykinin (Fig 6B). To simulate the bradykinin release from HMWK precursor, we designed a synthetic hexadecapeptide (Ac-SLMKRPPGFSPFRSSR-amide) designated extended bradykinin. This peptide was derived from the HMWK sequence to contain processing sites; *i*.*e*., the bradykinin sequence (RPPGFSPFR) was extended at the N- terminus by four additional HMWK residues (SLMK) and three residues on C-terminus (SSR). After incubation of this peptide with rSmSP2, the resulting fragments were separated by HPLC and the cleavage positions identified by mass spectrometry. The precursor was cleaved between Lys-Arg and Arg-Ser bonds thereby releasing bradykinin (Fig 6C). Synthetic bradykinin or kallidin (lysyl-bradykinin) were not cleaved by rSmSP2. Also, rSmSP2 degraded vasopressin, a nonapeptide hormone that increases arterial blood pressure by inducing vasoconstriction.

Next, we investigated whether living schistosomes produce a cleavage specificity similar to that of SmSP2 when incubating with vasopressin or extended bradykinin in culture. Adult schistosomes were incubated in the presence of the peptides and cleavage positions analyzed by mass spectrometry (Fig 6C). Both peptides were cleaved when added to the cultivation medium: vasopressin was inactivated by cleavage after penultimate Arg residue; extended bradykinin was cleaved to release bradykinin, however, cleavage precursors extended in N- or C-terminal direction were also identified. The fragmentation was significantly abolished in the presence of a serine protease specific inhibitor Pefabloc. The identified cleavage positions in the hormone sequences were identical with those obtained by *in vitro* fragmentation using rSmSP2.

The processing of human plasminogen, the precursor of the main fibrinolytic protease plasmin, by rSmSP2 was analyzed by SDS-PAGE and kinetic assay. Single chain plasminogen was completely converted into plasmin, consisting of a heavy chain and light chain). This processing was not the result of the self-activation as it only occurred in the presence of rSMSP2 (Fig 6D and 6E). SDS-PAGE analysis showed that SmSP2 processes single-chain tPA into its two-chain form; the cleavage was associated with a ten-fold increase in tPA activity (Fig 6F). tPA is the major physiological activator of plasminogen, thus the SmSP2 cleavage of single chain tPA into its fully active two-chain form would result in more efficient plasmin activation and in turn more effective fibrinolysis.

To conclude, SmSP2 cleaves several physiologically important blood proteins. Specifically, it processes the extracellular matrix and blood clot component, fibronectin, activates the major fibrinolytic enzyme, plasmin, and its activator, tPA, releases the vasodilatory peptide bradykinin from its HMWK precursor and processes the vasoconstrictory peptide vasopressin.

### SmSP2 is localized in the tegument, parenchyma and reproductive organs of adult schistosomes

**Fig 7:**
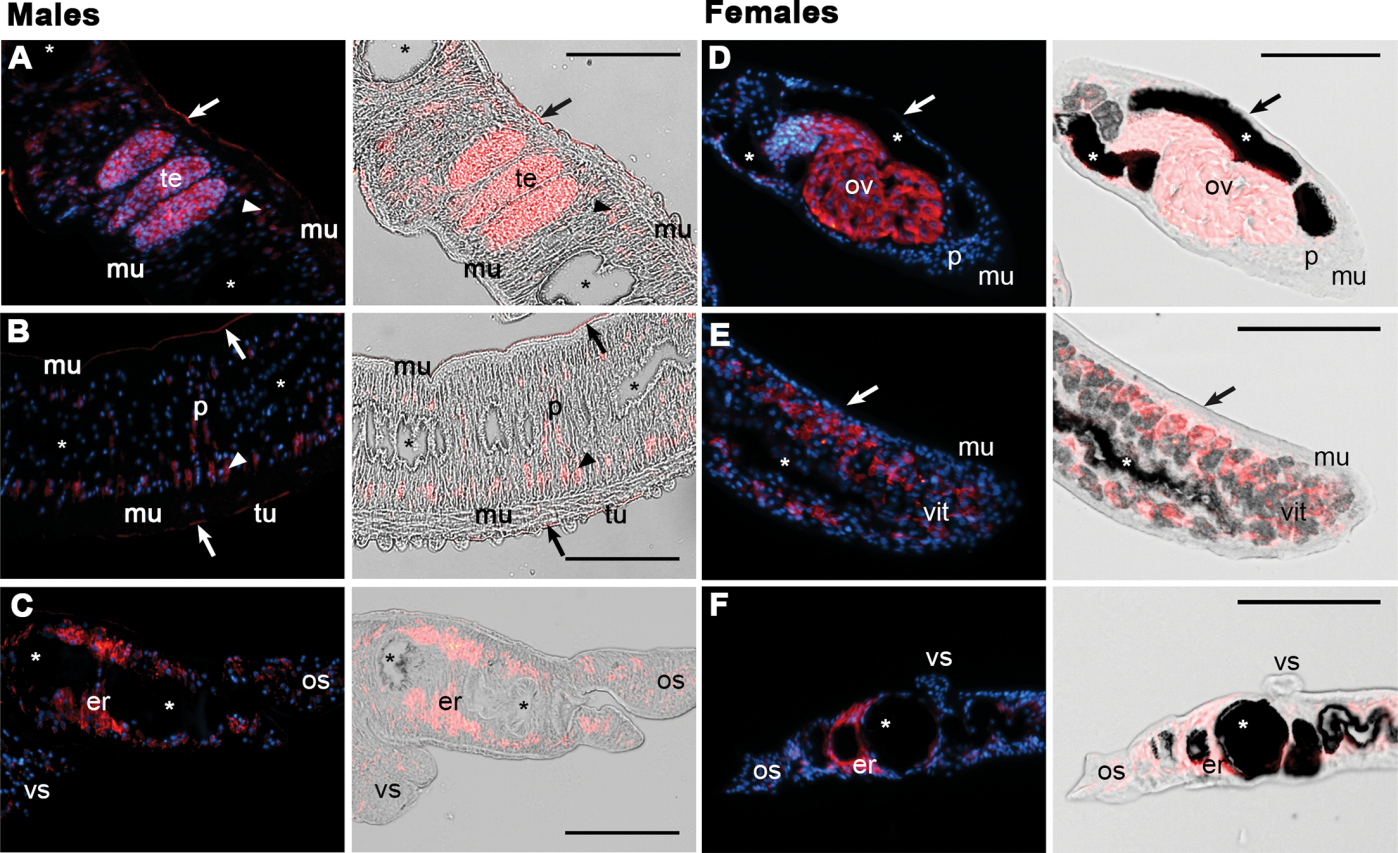
SmSP2 immunolocalization in sections of adult *S*. *mansoni*. Semi-thin sections of adult *S*. *mansoni* males (A to C) and females (D to F) were probed with an anti-SmSP2 IgG followed by reaction with an anti-rabbit Alexa 647-labeled secondary antibody (red). DAPI was used to label nuclear DNA (blue). The left columns show merged fluorescent channels; in the right columns, the signal is merged with differential interference contrast. A strong SmSP2 signal (red) was detected in both sexes in the parenchyma (p) and the esophageal region (er). A faint signal was noted in the ventral (vs) and oral suckers (os). No signal was detected in the gut (asterisks), muscle (mu) or tegumental tubercles (tu). In males, SmSP2 signal also appears in the tegument (arrowhead) in the tegumental membrane surface (arrow) and in the testes (te). In females, the signal is noted in the ovaries (ov) and vitellaria (vit). The scale bar represents 100 μm. A and D, reproductive organs; B and E, tegumental cells; C and F, head.

Indirect immunofluorescence microscopy on semi-thin sections using affinity purified polyclonal antibodies raised against rSmSP2 demonstrated that SmSP2 is expressed in distinct tissues of adult worms (Fig 7 and S5 Fig). In males, SmSP2 was observed in the parenchyma, tegument and in the tegumental surface membranes except the tubercles (for a detailed tegumental localization, see S5 Fig). In females, SmSP2 was observed in parenchyma but not in the tegument. In addition, intense staining was seen in the esophageal region of both genders, in the testes of males and in the ovaria and vitellaria of females (Fig 7). SmSP2 was absent from muscle, the tegumental tubercles, gastrodermis and gut lumen. Pre-immune serum was used as a negative control (S6 Fig) and only faint background fluorescence was detected.

## Discussion

Serine proteases of the S1 family are key factors of virulence for many parasitic helminths. They contribute to parasite invasion, migration, nutrition and reproduction, and facilitate adaption to and evasion of the host’s physiological and immune responses (for reviews see [12, 52]). Among serine proteases, most attention has been focused on *S*. *mansoni* cercarial elastases as these enzymes are implicated in tissue invasion and migration into the definitive mammalian hosts [13, 53]; however, information regarding other SmSPs is limited. Recently, we described the repertoire of non-cercarial elastase SmSPs by employing a series of genomic, transcriptomic, functional proteomic and phylogenetic approaches [23].

Here, we focus on SmSP2 as it is the most abundantly expressed SP in the *Schistosoma* blood-dwelling developmental stages [23]. The domain organization of SmSP2 is distantly reminiscent of the modular architecture of host blood-clotting serine proteases. Specifically, these enzymes contain a catalytic trypsin-like serine protease domain linked by disulfide bonds to an N-terminal multi-domain region that is involved in ligand-binding and protein-protein interactions [54]. SmSP2 also has unique features—an N-terminal region histidine stretch and a TSR-1 domain. TSR-1 domains mediate cell adhesion, protein-protein interactions and glycosaminoglycans binding [42, 43]. The histidine stretch may act as a metal binding site for divalent metal ions, as we demonstrated *in vitro* using metal-affinity chromatography to purify native SmSP2 from the adult schistosome homogenate. The TSR-1 domain is also present in the sequences of SmSP2 orthologs in other trematodes; cestodes retain only the TSR-1 domain while the histidine stretch is unique to trematodes (S3 Fig). Orthologous genes were not found in other organisms. Further research is needed to evaluate the exact function of the N-terminal domains of SmSP2.

SmSP2 orthologs (termed mastins) are present in S. *haematobium* and *S*. *japonicum*. For *S*. *haematobium* infections, mastin was identified in a protein array as an antigen targeted by a protective IgG1 immune response in those individuals with acquired resistance and, therefore, suggested as an attractive vaccine candidate [24]. This resistance is acquired after treatment with PZQ and the consequent exposure of parasite antigens to the host immune system [55]. SmSP2 orthologs are also present in the larval stage (cysticercus) of the cestodes, *Echinococcus granulosus* [56] and *Taenia solium* [57], and were identified as Antigen 5 excretory proteins (Ag5). Ag5 proteins are major components of cyst fluid and are used in a serodiagnostic test for cysticercosis [57, 58]. In comparison with SmSP2, the catalytic serine residue is replaced by threonine and Ag5 proteins show only marginal proteolytic activity [56, 57].

In adult *S*. *mansoni*, we demonstrate that SmSP2 is localized in the tegument. Treatment with PZQ causes tegumental damage [59, 60] and thereby exposes schistosome antigens, including SmSP2, to the host immune system [55]. The tegumental location may explain the recognition of SmSP2 by sera of PZQ-treated individuals [24]. Also, the localization of SmSP2 to the esophagus may indicate that the enzyme facilitates some aspect of nutrition during the ingestion of host proteins or is secreted. Indeed, we observed that SmSP2 is found in the ESP as was noted in *E*. *granulosus* previously for Ag5 [58, 61]. In addition, SmSP2 and its *S*. *japonicum* mastin ortholog were recently proteomically identified in exosome-like vesicles that are secreted by parasite and putatively modulate host-parasite interactions [62–64]. Finally, apart from a potential extracorporeal function, the protease domain of SmSP2 was localized to a number of internal tissues, including the ovaries, testes, muscle and parenchyma, suggesting a variety of functional roles.

To enzymatically characterize SmSP2, the protease domain was heterologously expressed in *P*. *pastoris* and purified as an active protease. SmSP2 was subjected to a range of biochemical analyses to determine its substrate and inhibitory specificity. SmSP2 was classified as a trypsin-like enzyme as it cleaves various peptide substrates in an endopeptidolytic mode at the carboxyl terminus of Arg or Lys residues and was inhibited by inhibitors targeting trypsin-like proteases such as leupeptin, benzamidine and antipain [65]. Consistent with this, systematic cleavage specificity analysis with the positional-scanning and multiplex substrate libraries revealed a preference for basic amino acids at P1. In agreement with the cleavage specificities, the homology model of SmSP2 reveals that the S1 binding pocket has an architecture analogous to vertebrate trypsins, including the critical Asp441 residue that defines the preference for basic P1 residues [45].

Based on homology modeling, the SmSP2 protease domain contains a large 43 residue-long insertion (at position 358) which is a unique structural feature of SmSP2 and its trematode orthologs not present in other S1 family proteases. This insertion is localized in the vicinity of the active site on the loop D (nomenclature according to Perona and Craik [48]). We demonstrate that SmSP2 performs limited proteolysis to process a number of physiologically- relevant host proteins: it degrades fibronectin, activates plasmin and tPA and releases bradykinin from its precursor HMWK. SmSP2 is incapable of cleaving macromolecular substrates such as Hb, BSA and IgG, which, for example, are cleaved by the gut-associated cysteine and aspartic proteases [15, 16]. SmSP2 has a more narrow substrate specificity than trypsin, the S1 family archetypal protease. In multiplex cleavage assays, trypsin cleaved more substrates than SmSP2. It resulted in similar number of cleavages produced by trypsin in 15 min whereas 4 h was required by SmSP2. A plausible explanation is that the B and D loop-insertions on SmSP2 limit the access of substrates to the active site resulting in the selective processing of conformationally compatible peptides. However, the insertion-D does not directly occlude the active site subsites as this would confer an exopeptidase activity that is not observed for SmSP2.

Adult schistosomes can survive for decades in the host [66]. It is thought that these large intravascular parasites manipulate the complex hemostatic system of the host at different levels via bioactive molecules in the ESP or on the tegument [67]. However, the detailed molecular processes underlying these survival mechanisms are not fully understood. Our work demonstrates that SmSP2 is present in both the ESP and tegument. We show that SmSP2 possesses a kallikrein-like activity as it cleaves the plasma protein kininogen to generate the peptide hormone, bradykinin. Bradykinin is a potent vasodilator that decreases blood pressure and increases vascular permeability [68]. Bradykinin also exerts anti-thrombogenic, anti-proliferative and anti-fibrogenic effects [69]. In a recent report, we demonstrated that living schistosomes cleave bradykinin and angiotensin I (converting this vasoconstrictor to the vasodilatory angiotensin-(1–7)), and that the tegumental, S9 serine protease, SmPOP, is involved in that processing [37]. Moreover, SmSP2 inactivates vasopressin, a hormone that increases arterial blood pressure by inducing vasoconstriction. It is, therefore, possible that both SmPOP and SmSP2 modulate the parasite's local vascular environment to the parasite’s benefit during its residence and movement in the host’s blood vessels. Additionally, we show that the whole schistosome parasite can cleave the vasopressin and release bradykinin from its synthetic precursor, when co-incubated *in vitro*. The activity is due to a serine protease(s) (possibly SmSP2) that is most abundantly expressed serine protease in adult schistosome [23] as indicated by mRNA expression levels, mass spectrometry and specific inhibition by a serine protease inhibitor.

**Fig 8:**
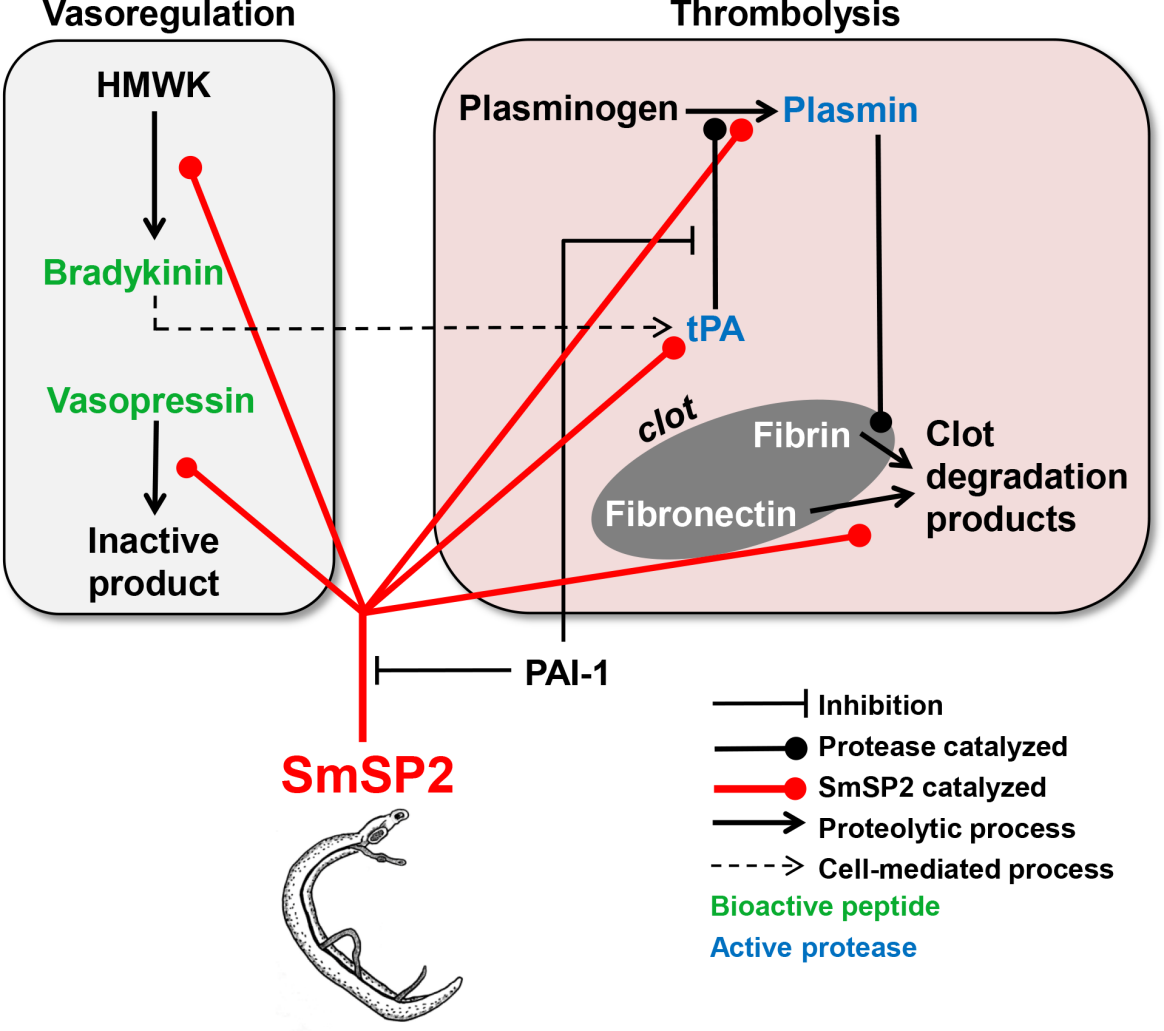
Model of how SmSP2 may interfere with the host’s hemostatic system. SmSP2, secreted from adult schistosomes or localized at the surface, stimulates the degradation of blood clots (thrombolysis panel) by (i) activation of two critical components of the fibrinolytic system, tissue plasminogen activator (tPA) and plasminogen, and (ii) direct degradation of the blood-clot component, fibronectin. SmSP2 modulates vascular tone (vasoregulation panel) by processing bioactive peptide hormones. (i) It releases the vasodilatory bradykinin from kininogen (HMWK) and (ii) degrades the vasoconstrictory peptide, vasopressin. Bradykinin may stimulate the release of tPA from vascular endothelial cells (dashed line) which would promote fibrinolysis. SmSP2 may be regulated by plasminogen activator inhibitor-1 (PAI-1) that inhibits SmSP2 (Table 1).

Due to their large size, adult schistosomes may alter or disrupt normal blood flow, and damage the endothelium, which could lead to platelet activation and subsequently blood coagulation [67]. However, blood clots are not observed around the parasites residing in the host blood vessels and several mechanisms have been proposed with which schistosomes inhibit blood clot formation and/or promote blood clot lysis [70]. Physiologically, the latter process involves proteolytic degradation of fibrin that is mediated by plasmin. This central protease in the fibrinolytic system is generated from its zymogen, plasminogen (PLG), by, for example, tissue plasminogen activator (tPA). Based on our *in vitro* results, we propose here a new and complex mechanism with which schistosomes employ SmSP2 to promote fibrinolysis at multiple levels (Fig 8): 1) SmSP2 activates PLG to plasmin; this action can be accelerated by presentation of PLG on tegumental receptors (*e*.*g*., enolase [71] of schistosomes); 2) SmSP2 processes the single-chain tPA to the more potent double-chain tPA form [72] which would then cause enhanced plasmin activation, 3) SmSP2 produces bradykinin that is known to stimulate the release of tPA from the vascular endothelium [73]; and 4) SmSP2 directly degrades the blood clot component fibronectin, which has also been recently shown for a tegumental calpain [74].

To conclude, we have expressed, and biochemical and functionally characterized the multi-domain serine protease, SmSP2. The protease is a putative anti-hemostatic peculiar to platyhelminths, which are the only pathogens to express SmSP2 orthologs. Further research is needed to evaluate the role of SmSP2 (and orthologs) in modulating host-parasite interactions and its potential as a drug or vaccine candidate.

## Supporting information

S1 Fig Multiple sequence alignment of the TSR-1 domain of SmSP2 with selected TSR-1 domains of human proteins. Sequences are: TSP-1-1-3—thrombospondin-1 (TSP) type-1 domains 1, 2 and 3 (Uniprot accession number: P07996), properdin-TSR1-6—properdin thrombospondin type-1 domains 1–6 (P27918), ADAMTS13 (Q76LX8), and spondin-TSP-1—spondin-1 thrombospondin type-1 domain 1 (Q9HCB6). Cys residues are highlighted in yellow, tryptophan substituents forming the W layer are in blue, and amino acid residues forming the R layers are in green.
(TIF)

S2 Fig Multiple sequence alignment of the SmSP2 protease domain with catalytic domains of selected human and bovine S1 family proteases. Human proteases: mannan-binding lectin serine protease 1 (MASP-1, Uniprot accession number: P48740), tissue plasminogen activator (tPA, P00750), urokinase plasminogen activator (uPA, P00749), plasmin (P00747), kallikrein 1 (P06870) and matriptase (MTSP-1, Q9Y5Y6). Bovine proteases: cationic trypsin (P00760) and chymotrypsin A (P00766). The catalytic triad residues (His, Asp, Ser) are red-boxed; the critical Asp residue in the S1 subsite that accounts for trypsin-like activity is green-boxed. Cys residues that are predicted to form disulfide bonds are indicated by the same color, cyan Cys form interchain disulfide bond with domains not included in the alignment. Residues that are shared between sequences are shaded in grey. Residues forming SmSP2 insertion-222, 251, and 358 are underlined. The upper line numbering is according to SmSP2, the lower line numbering according to bovine chymotrypsinogen.
(TIF)

S3 Fig Multiple sequence alignment of SmSP2 with orthologs from other platyhelminth parasites. Trematode sequences: *Schistosoma*. *japonicum* (GenBank: AAW24683.1), *Schistosoma haematobium* (XP_012796372.1), *Fasciola hepatica* (sequence identified in the transcriptome database (Young et al. (2010), Biotechnol Adv 28, 222–231), *Opisthorchis viverrini* (XP_009167273.1) and *Clonorchis sinensis* (GAA32831.2). Cestode sequences: *Hymenolepis microstoma* (CDS25513.1), *Echinococcus multiocularis* (CDI97096.1), *Echinococcus granulosus* (EUB58856.1) and *Taenia solium* (ADP89566.1). Predicted signal sequences are in blue, histidine residues in the N-terminal region are in purple and the TSR-1 domain is in green. The catalytic triad residues (His, Asp, Ser) are red-boxed, the critical Asp residue in S1 subsite that accounts for trypsin-like activity is green-boxed. Cys residues in the TSR-1 domain are highlighted in yellow and Cys residues in the protease domain are in cyan.
(TIF)

S4 Fig Binding of native SmSP2 to a Ni^2+^-ion affinity column. A protein extract of adult schistosomes (Extract) was applied to a HiTrap IMAC FF column containing immobilized Ni^2+^ ions and native SmSP2 eluted using 0.5 M imidazole. The extract, unbound material (FT) and eluted material (Elution) were resolved by SDS-PAGE, electrophoretically transferred onto a PVDF membrane and visualized by anti-rSmSP2 IgG.
(TIF)

S5 Fig Detailed micrograph of SmSP2 localization in the tegument of adult male *S*. *mansoni*. The tissue section was probed with anti-SmSP2 IgG followed by an anti-rabbit IgG Alexa 594-labeled secondary antibody (red). DAPI was used to label the nuclear DNA (blue). The left image shows merged fluorescent channels; on the right, schematic depiction of the adult schistosome surface; sm—surface membrane, tg—tegument, mu—muscle, tc—tegumental cell (cyton), pa—parenchym.
(TIF)

S6 Fig Pre-immune serum is not reactive. As a negative control, semi-thin sections of adult S. mansoni males and females were probed with a pre-immune serum (A-F) followed by reaction with an anti-rabbit IgG Alexa 647-labeled secondary antibody (red). DAPI was used to label nuclear DNA (blue). The first and third columns show merged fluorescent channels; in the second and fourth columns, the signal is merged with differential interference contrast.
(TIF)
