# Supplementary figures and images for: SmSP2: A serine protease secreted by the blood fluke pathogen Schistosoma mansoni with anti-hemostatic properties
Source: PLoS Negl Trop Dis. 2018 Apr 20;12(4):e0006446. doi: 10.1371/journal.pntd.0006446 (PMC5931690; doi:10.1371/journal.pntd.0006446)

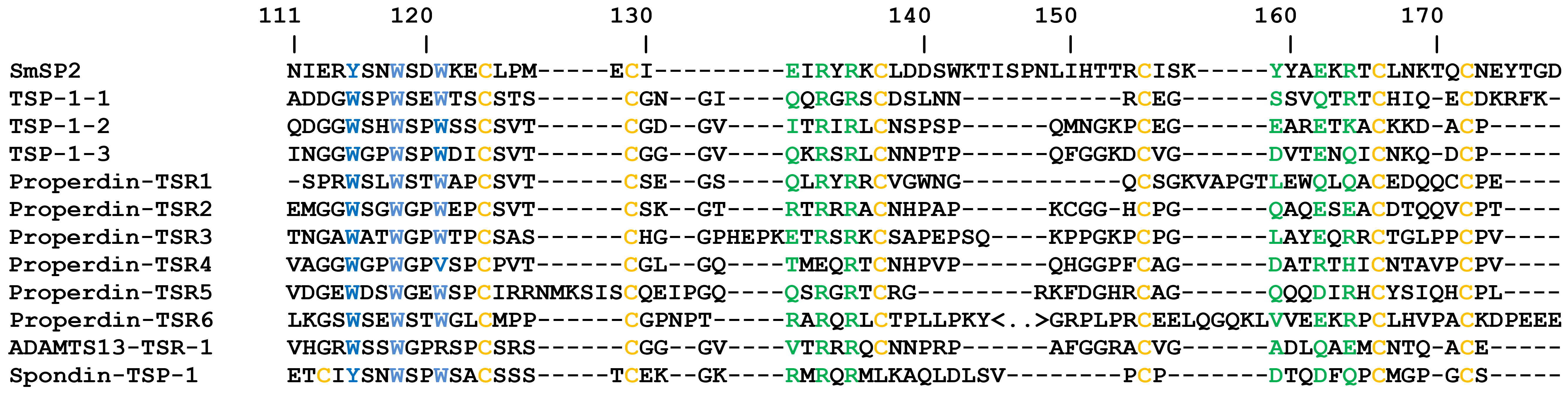

Supplement: S1 Fig — Sequences are: TSP-1-1-3—thrombospondin-1 (TSP) type-1 domains 1, 2 and 3 (Uniprot accession number: P07996), properdin-TSR1-6—properdin thrombospondin type-1 domains 1–6 (P27918), ADAMTS13 (Q76LX8), and spondin-TSP-1—spondin-1 thrombospondin type-1 domain 1 (Q9HCB6). Cys residues are highlighted in yellow, tryptophan substituents forming the W layer are in blue, and amino acid residues forming the R layers are in green. (TIF) [file pntd.0006446.s001.tif]

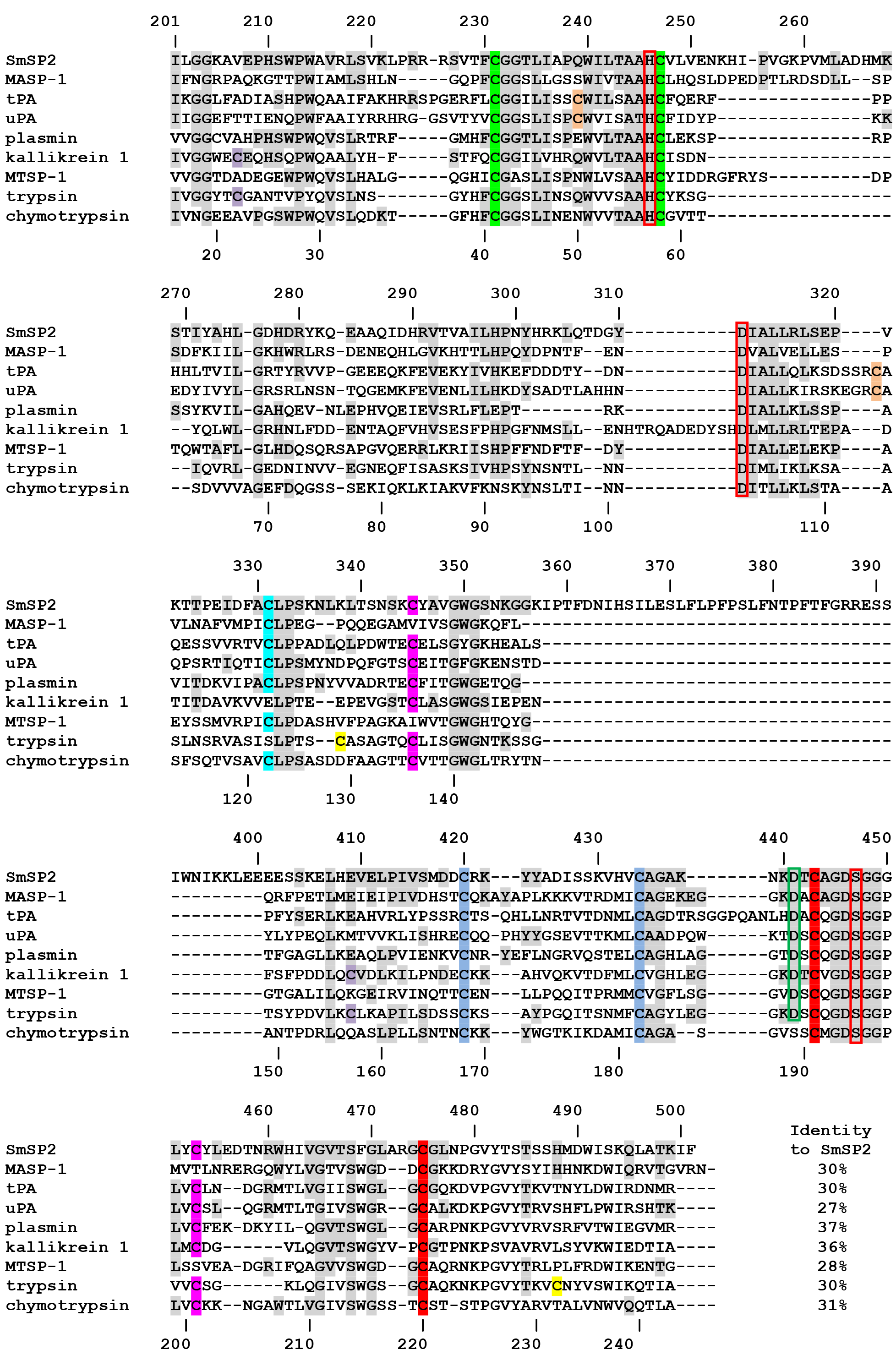

Supplement: S2 Fig — Human proteases: mannan-binding lectin serine protease 1 (MASP-1, Uniprot accession number: P48740), tissue plasminogen activator (tPA, P00750), urokinase plasminogen activator (uPA, P00749), plasmin (P00747), kallikrein 1 (P06870) and matriptase (MTSP-1, Q9Y5Y6). Bovine proteases: cationic trypsin (P00760) and chymotrypsin A (P00766). The catalytic triad residues (His, Asp, Ser) are red-boxed; the critical Asp residue in the S1 subsite that accounts for trypsin-like activity is green-boxed. Cys residues that are predicted to form disulfide bonds are indicated by the same color, cyan Cys form interchain disulfide bond with domains not included in the alignment. Residues that are shared between sequences are shaded in grey. Residues forming SmSP2 insertion-222, 251, and 358 are underlined. The upper line numbering is according to SmSP2, the lower line numbering according to bovine chymotrypsinogen. (TIF) [file pntd.0006446.s002.tif]

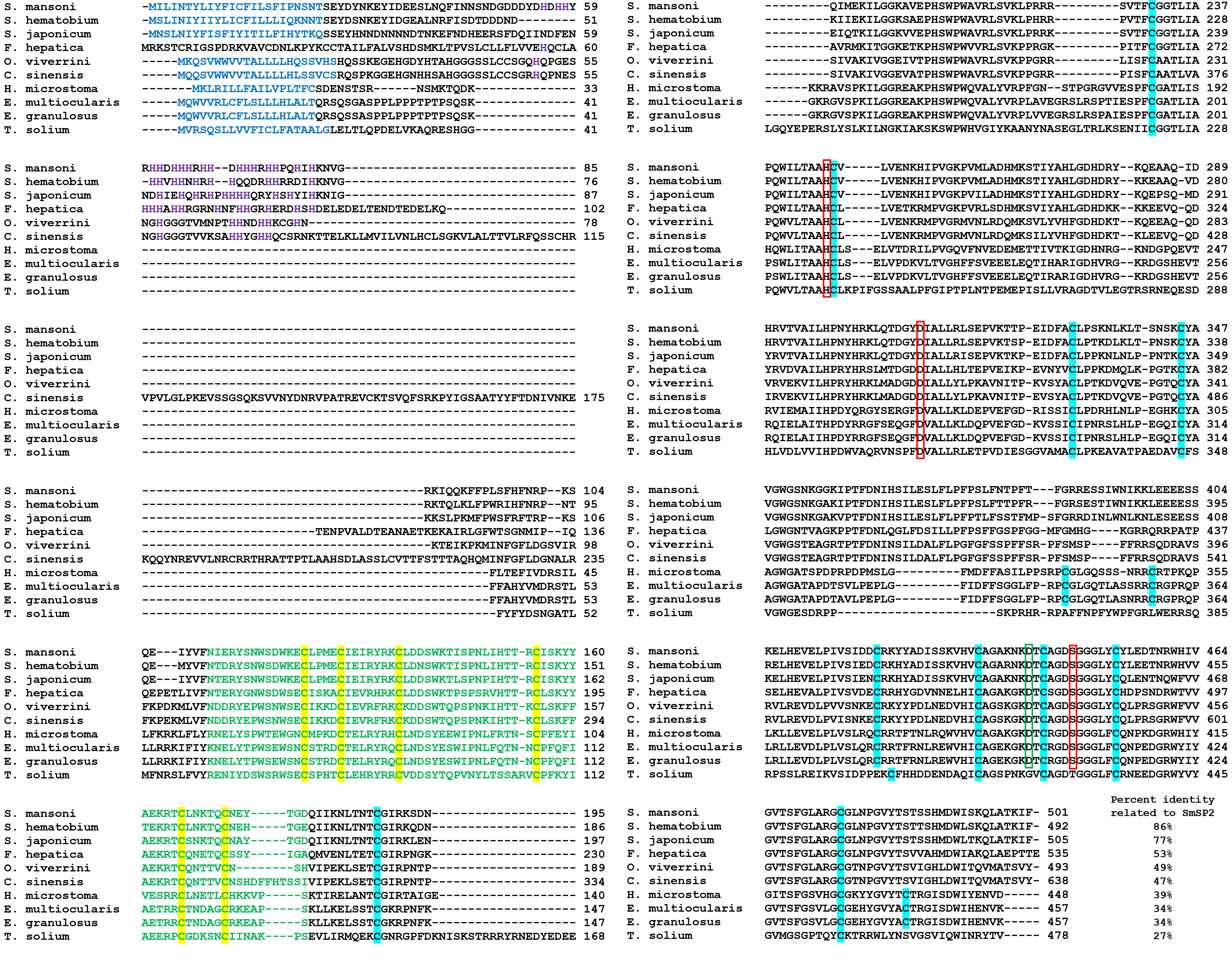

Supplement: S3 Fig — Trematode sequences: Schistosoma. japonicum (GenBank: AAW24683.1), Schistosoma haematobium (XP_012796372.1), Fasciola hepatica (sequence identified in the transcriptome database (Young et al. (2010), Biotechnol Adv 28, 222–231), Opisthorchis viverrini (XP_009167273.1) and Clonorchis sinensis (GAA32831.2). Cestode sequences: Hymenolepis microstoma (CDS25513.1), Echinococcus multiocularis (CDI97096.1), Echinococcus granulosus (EUB58856.1) and Taenia solium (ADP89566.1). Predicted signal sequences are in blue, histidine residues in the N-terminal region are in purple and the TSR-1 domain is in green. The catalytic triad residues (His, Asp, Ser) are red-boxed, the critical Asp residue in S1 subsite that accounts for trypsin-like activity is green-boxed. Cys residues in the TSR-1 domain are highlighted in yellow and Cys residues in the protease domain are in cyan. (TIF) [file pntd.0006446.s003.tif]

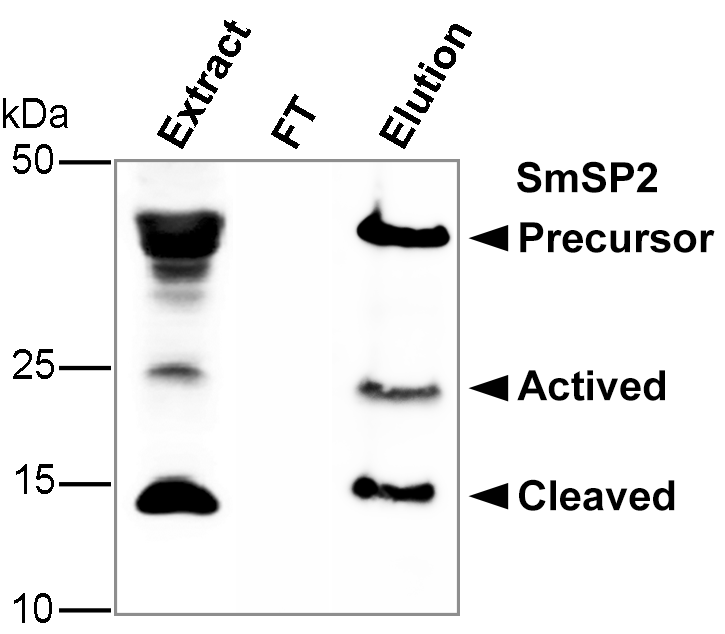

Supplement: S4 Fig — A protein extract of adult schistosomes (Extract) was applied to a HiTrap IMAC FF column containing immobilized Ni2+ ions and native SmSP2 eluted using 0.5 M imidazole. The extract, unbound material (FT) and eluted material (Elution) were resolved by SDS-PAGE, electrophoretically transferred onto a PVDF membrane and visualized by anti-rSmSP2 IgG. (TIF) [file pntd.0006446.s004.tif]

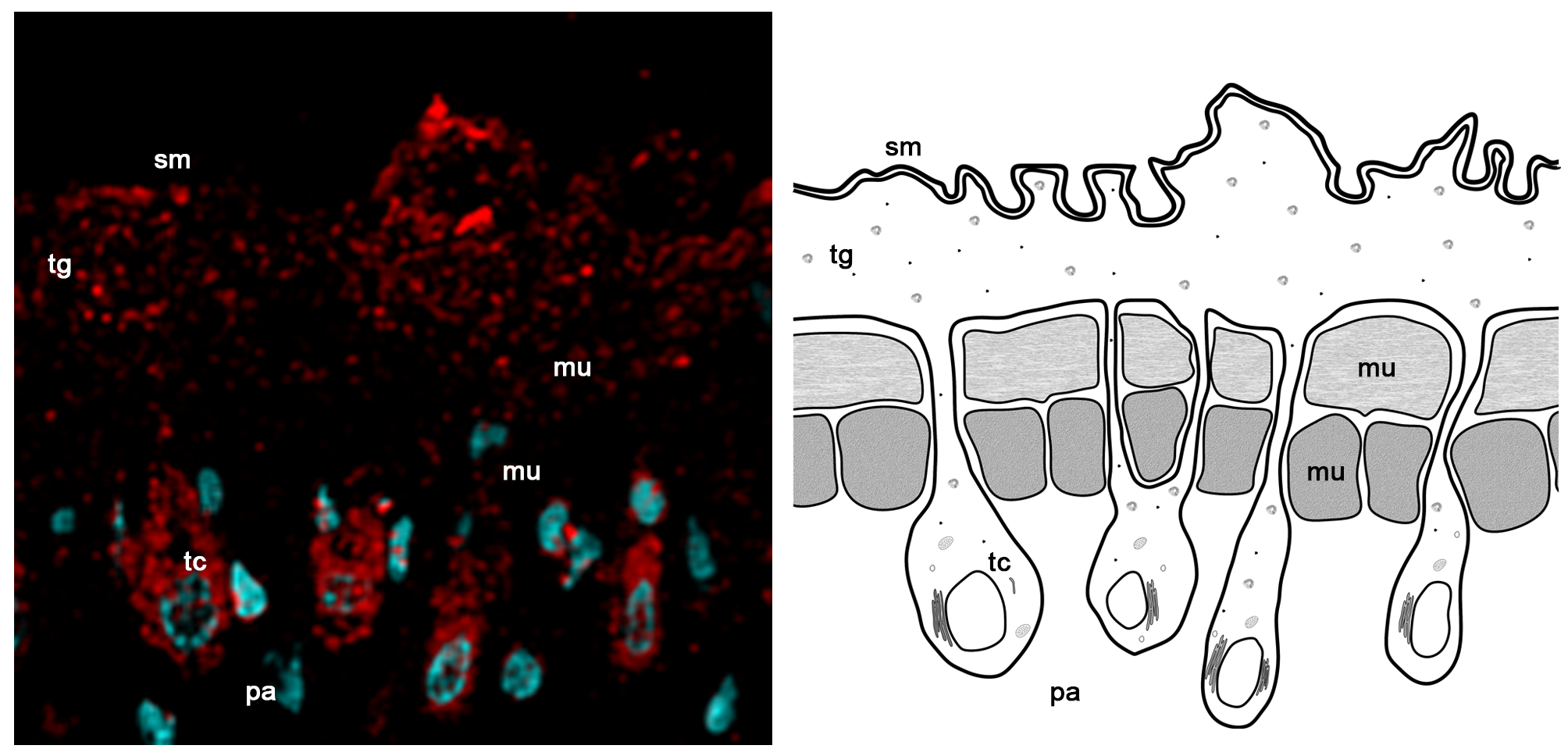

Supplement: S5 Fig — The tissue section was probed with anti-SmSP2 IgG followed by an anti-rabbit IgG Alexa 594-labeled secondary antibody (red). DAPI was used to label the nuclear DNA (blue). The left image shows merged fluorescent channels; on the right, schematic depiction of the adult schistosome surface; sm—surface membrane, tg—tegument, mu—muscle, tc—tegumental cell (cyton), pa—parenchym. (TIF) [file pntd.0006446.s005.tif]

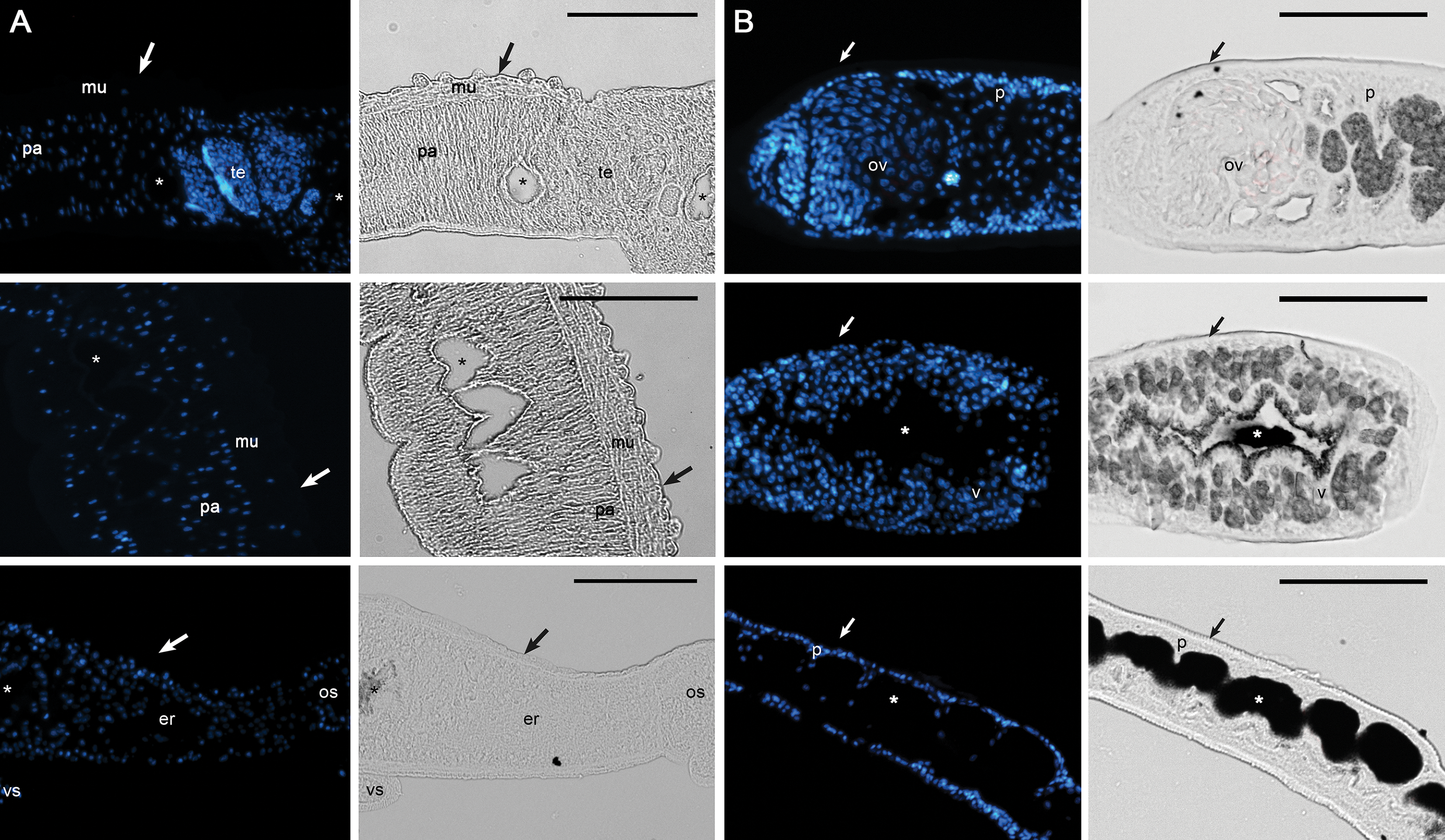

Supplement: S6 Fig — As a negative control, semi-thin sections of adult S. mansoni males and females were probed with a pre-immune serum (A-F) followed by reaction with an anti-rabbit IgG Alexa 647-labeled secondary antibody (red). DAPI was used to label nuclear DNA (blue). The first and third columns show merged fluorescent channels; in the second and fourth columns, the signal is merged with differential interference contrast. (TIF) [file pntd.0006446.s006.tif]
